# CRISPR‐MI and scRNA‐Seq Reveal TREM2's Function in Monocyte Infiltration and Macrophage Apoptosis During Abdominal Aortic Aneurysm Development

**DOI:** 10.1002/advs.202412227

**Published:** 2025-10-07

**Authors:** Haocheng Lu, Chao Xue, Yang Zhao, Jinjian Sun, Changzhi Zhao, Xu Zhang, Guizhen Zhao, Yaozhong Liu, Hongyu Liu, Yongjie Deng, Ying Wang, Chi Zhang, Yingjie Liu, Linjun Zeng, Ying Yang, Bolun Li, Shusi Ding, Linkang Zhou, Henry Kuang, Zanxin Wang, Wenhao Ju, Haihuan Lin, Jie Lin, Yanhong Guo, Lin Chang, Hongmei Zhao, Jing Wang, Jiandie Lin, Lemin Zheng, Y. Eugene Chen, Jifeng Zhang

**Affiliations:** ^1^ Department of Pharmacology Joint Laboratory of Guangdong‐Hong Kong Universities for Vascular Homeostasis and Diseases School of Medicine Southern University of Science and Technology Shenzhen Guangdong 518033 P. R. China; ^2^ Department of Internal Medicine Frankel Cardiovascular Center University of Michigan, Medical Center Ann Arbor MI 48105 USA; ^3^ Department of Internal Medicine Rochester General Hospital Rochester NY 14621 USA; ^4^ Department of General &Vascular Surgery Xiangya Hospital Central South University Changsha Hunan 410008 P. R. China; ^5^ Yazhouwan National Laboratory Sanya 572024 P. R. China; ^6^ Laboratory of Translational Genetics Division of Cancer Epidemiology & Genetics National Cancer Institute Bethesda MD 20892 USA; ^7^ Beijing Tiantan Hospital, China National Clinical Research Center for Neurological Diseases, Advanced Innovation Center for Human Brain Protection Capital Medical University Beijing 100050 China; ^8^ Life Sciences Institute University of Michigan Ann Arbor MI 48109 USA; ^9^ Department of Cardiovascular Surgery The University of Hong Kong‐Shenzhen Hospital Guangdong 518031 P. R. China; ^10^ Department of Structural Heart Disease National Center for Cardiovascular Disease China & Fuwai Hospital Chinese Academy of Medical Sciences & Peking Union Medical College Beijing 100037 P. R. China; ^11^ National Clinical Research Center for Cardiovascular Diseases Fuwai Hospital Chinese Academy of Medical Sciences Beijing 100037 P. R. China; ^12^ Cardiology Department The First Affiliated Hospital of Wenzhou Medical University 325000 Wenzhou P. R. China; ^13^ State Key Laboratory of Respiratory Health and Multimorbidity, Institute of Basic Medical Sciences Chinese Academy of Medical Sciences School of Basic Medicine Peking Union Medical College Beijing 100050 P. R. China; ^14^ Department of Cell and Developmental Biology University of Michigan Medical Center Ann Arbor MI 48109 USA; ^15^ The Institute of Cardiovascular Sciences School of Basic Medical Sciences, Key Laboratory of Molecular Cardiovascular Science of Ministry of Education NHC Key Laboratory of Cardiovascular Molecular Biology and Regulatory Peptides Beijing Key Laboratory of Cardiovascular Receptors Research Health Science Center Peking University Beijing 100191 P. R. China

**Keywords:** abdominal aortic aneurysm, apoptosis, CRISPR screen, inflammation, macrophage

## Abstract

Abdominal aortic aneurysm (AAA) is a life‐threatening aortic disease without effective medication. The infiltration of monocytes into the aortic wall is critical for AAA development, but the genes and pathways regulating this process remain to be elucidated. A novel method is developed for in vivo genome‐wide CRISPR/Cas9 screening of monocyte infiltration (CRISPR‐MI). By combining CRISPR‐MI with single‐cell RNA sequencing (scRNA‐Seq), this study finds that Triggering receptor expressed on myeloid cells 2 (*Trem2)* is a negative regulator of monocyte infiltration into the aortic wall in early AAA induction. *Trem2* knockout (KO) increases the expression of adhesion molecules, chemotactic receptors, and cytokines in monocytes. *Trem2* KO promotes monocyte adhesion and migration in vitro and increases monocyte infiltration into the aortic wall in vivo. However, *Trem2* KO attenuates AAA development because of prominent macrophage death at the late stage. In conclusion, CRISPR‐MI is a powerful tool for studying genes underlying monocyte infiltration in disease conditions in vivo. These findings reveal a dichotomous role of *Trem2* in monocyte recruitment and macrophage survival during AAA.

## Graphical abstract

A method is developed for conducting genome‐wide CRISPR/Cas9 screening of monocyte infiltration in vivo (CRISPR‐MI) that is easily adaptable across a variety of disease models. Through the combination of CRISPR‐MI and scRNA‐Seq, this study discovers that Trem2 is a key regulator of early monocyte infiltration in abdominal aortic aneurysm (AAA).

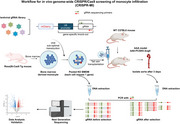

## 1 Introduction

Abdominal aortic aneurysm (AAA) refers to the local dilation of the abdominal aorta with a diameter larger than 3 cm or more than 50% larger than the normal size. AAA is typically asymptomatic, but it can lead to dissection, rupture, or other life‐threatening complications.^[^ ^1^ ^]^ Aortic aneurysm is the second most common aortic disease following atherosclerosis and accounts for the ninth‐leading cause of death overall.^[^ ^2^ ^]^ The prevalence of AAA in Western populations has been reported to be ≈1%, but its incidence increases with age.^[^ ^3^, ^4^ ^]^ The prevalence of AAA in individuals aged 75 to 84 years old reaches 12.5%,^[^ ^4^ ^]^ which has a significant impact on public health.

The aorta can be divided into the thoracic aorta and abdominal aorta, and the AAA incidence is about three times that of the thoracic aortic aneurysm.^[^ ^2^ ^]^ The risk factors of AAA include smoking, male sex, increased age, hypertension, dyslipidemia, and obesity.^[^ ^2^ ^]^ While the pathogenesis of AAA remains incompletely understood, its pathological features are characterized by prominent inflammation, loss of vascular smooth muscle cells (VSMCs), and degradation of extracellular matrix (ECM). The therapeutic intervention of AAA mainly relies on open surgery or endovascular aneurysm repair.^[^ ^5^ ^]^ Although the last two decades have witnessed major advancements in AAA surgical and endovascular repair, medication for AAA is still lacking, highlighting the need for a deeper study into the molecular mechanisms of AAA.

The inflammatory response has a crucial role in the AAA development and sequential vascular wall remodeling. Inflammatory cell infiltration into the aortic wall and adventitia is observed in both human^[^ ^6^, ^7^ ^]^ and murine AAA.^[^ ^8^ ^]^ Accumulation of macrophages in the aortic wall contributes to local inflammation by secreting various inflammatory cytokines and chemokines.^[^ ^9^ ^]^ Macrophage also influences the vascular smooth muscle phenotypical switch^[^ ^10^ ^]^ and produces matrix‐degrading proteinases. Based on their origin, aortic macrophages can be classified into two types. One is resident macrophages, which seed into the aortic intima at birth and are maintained by self‐renew^[^ ^11^, ^12^ ^]^; The other one is bone‐marrow‐derived macrophages recruited from the circulating monocyte pool.^[^ ^13^, ^14^ ^]^ In the healthy aorta, the blood monocytes contribute minimally to aortic macrophage, but in the early phase of Angiotensin II (AngII) infusion (<10 days), the increase of macrophages is largely driven by the recruitment of blood monocytes.^[^ ^14^ ^]^ This early phase of inflammation is critical for arterial injury, and intervening in this early phase of inflammation may help prevent or mitigate the development of aortic diseases.

In humans, circulating monocytes are heterogeneous leukocyte populations, including classical monocytes (CD14^++^CD16^−^), non‐classical monocytes (CD14^+^CD16^++^), and intermediate monocytes (CD14^++^CD16^+^).^[^ ^15^, ^16^ ^]^ In mice, Ly6C^high^ monocytes are similar to human classical monocytes, and Ly6C^low^ monocytes have functions similar to human non‐classical monocytes.^[^ ^17^ ^]^ Non‐classical monocytes normally patrol along the vascular endothelium, participating in vascular homeostasis and immune surveillance.^[^ ^18^ ^]^ Instead, classical monocytes are activated and infiltrated into inflammatory sites, which rely on CCR2.^[^ ^19^ ^]^ Infiltrated monocytes differentiated into macrophages in the tissue, and the macrophage phenotype is determined by both their origin and microenvironment.^[^ ^20^ ^]^ In mice, upon microbial stimulation, Ly6C^high^ monocytes give rise to iNOS‐positive (M1‐like) macrophages.^[^ ^21^ ^]^ On the other hand, during tissue repair, non‐classical monocytes extravasate and become alternatively activated (M2‐like) macrophages.^[^ ^22^ ^]^ The monocyte‐to‐macrophage differentiation process in humans is less understood and may differ in different diseases.^[^ ^23^, ^24^ ^]^

The infiltration of monocytes into the aortic wall is a multistep process, including tethering and rolling, arrest and firm adhesion, and transendothelial migration.^[^ ^25^ ^]^ First, the initial tethering and rolling of monocytes is mediated by selectins (L‐selectin on monocytes, and P‐selectin and E‐selectin on endothelial cells). Next, the integrins on monocytes are activated by various cytokines. The activated LFA1 and VLA4 interact with ICAM1 and VCAM on the ECs, respectively, resulting in the establishment of firm adhesion. Finally, the activated integrins on monocytes (LFA1, VLA4, and Mac1) interact with JAM family proteins (JAM‐A, JAM‐B, JAM‐C) on ECs to facilitate the migration through tight junctions, with the help of homophilic proteins, including PECAM‐1 and CD99.^[^ ^25^, ^26^, ^27^ ^]^ Several chemokine receptors are implicated in the monocyte recruitment process, including CCL2‐CCR2, CX3CL1–CX3CR1, and CCL3/CCL5‐CCR1/CCR5.^[^ ^28^ ^]^ However, pharmacological agents that selectively target monocyte infiltration in treating cardiovascular diseases are still lacking, and further understanding of this complicated process is required.

Clustered regularly interspaced short palindromic repeats (CRISPR) screening is a powerful tool to investigate biological processes. The efficient and flexible CRISPR‐Cas9 genome editing system can be utilized for forward genetic screening, permitting unbiased high‐throughput screen. Compared with traditional shRNA‐based screens, CRISPR‐Cas9 is able to completely knock out the gene and incurs fewer off‐target events.^[^ ^29^ ^]^ In the CRISPR‐Cas9 screen, the mutant cell pool is generated by the introduction of Cas9 and sgRNA library into cells, followed by positive/negative selection. sgRNAs enriched or depleted after selection are identified by high‐throughput sequencing. sgRNAs that are enriched or depleted correspond to genes that affect the cellular capability to pass through the selection.^[^ ^30^, ^31^ ^]^ The CRISPR‐Cas9 screen requires a large pool of mutant cells and a reliable selection procedure, which can usually only be performed in vitro or in xenograft models.^[^ ^32^, ^33^, ^34^ ^]^ This disadvantage limits the application of CRISPR‐Cas9 screen in cardiovascular diseases, as CVD pathogenesis involves interactions between multiple cell types, and it is difficult to recapitulate the microenvironment in the in vitro system. To overcome this challenge, we developed an unbiased genome‐wide CRISPR‐Cas9 Screen for Monocyte Infiltration in vivo (CRISPR‐MI) and employed this new method in a mouse AAA model.

With CRISPR‐MI, we identified Triggering receptor expressed on myeloid cells 2 (*Trem2)* as a negative regulator of monocyte extravasation. Serum soluble TREM2 (sTREM2) has been reported to be associated with coronary heart diseases.^[^ ^35^, ^36^, ^37^ ^]^ In the deoxycorticosterone acetate (DOCA)‐salt hypertension mouse model, *Trem2* knockout (KO) increased cardiac hypertrophy and diastolic dysfunction.^[^ ^38^ ^]^ *Trem2* KO aggravated myocardial ischemia (MI) injury and decreased heart function.^[^ ^39^ ^]^ The role of *Trem2* in atherosclerosis is still controversial: two studies reported that *Trem2* KO decreased atherosclerotic plaque,^[^ ^40^, ^41^ ^]^ while another study reported that *Trem2* KO did not affect plaque size but increased the necrotic core area.^[^ ^42^ ^]^ In mouse AAA models, single‐cell RNA sequencing (scRNA‐Seq) revealed the enrichment of *Trem2* ^high^ macrophage populations in disease tissue, suggesting a positive association of *Trem2* expression with aneurysm pathology.^[^ ^43^ ^]^ Whether TREM2 plays a causative role in AAA development, and the biological functions of TREM2 in disease‐related monocytes and macrophages, remain to be elucidated.

In our study, we found that sTREM2 level is increased in the plasma in both AAA mice and patients. *Trem2* KO monocytes expressed higher levels of adhesion molecules and inflammatory cytokines. The infiltration of monocytes into the aorta after AngII infusion is significantly increased in *Trem2* KO mice. Surprisingly, we found that infiltrated *Trem2* KO macrophages underwent significantly more apoptosis in the aortic wall, leading to fewer AAA development in *Trem2* KO mice. Our study demonstrated that *Trem2* is both important for maintaining monocyte quiescence in circulation and crucial for macrophage survival in the aorta. This dichotomous role of *Trem2* may help to understand the controversial results observed in *Trem2* KO mice in various disease models and studies.

## 2 Results

### 2.1 Development of an In Vivo Genome‐Wide CRISPR Cas9 Screen for Monocyte Infiltration (CRISPR‐MI)

There are three challenges we encountered in establishing the in vivo CRISPR screen method. 1) To cover the genome‐wide screen, we need to get large amounts of cells to generate a mutant cell pool; 2) We need to optimize the sgRNA library transfection efficiency in primary cells; 3) We need to maximize the genome editing efficiency in primary cells.

**Figure 1:**
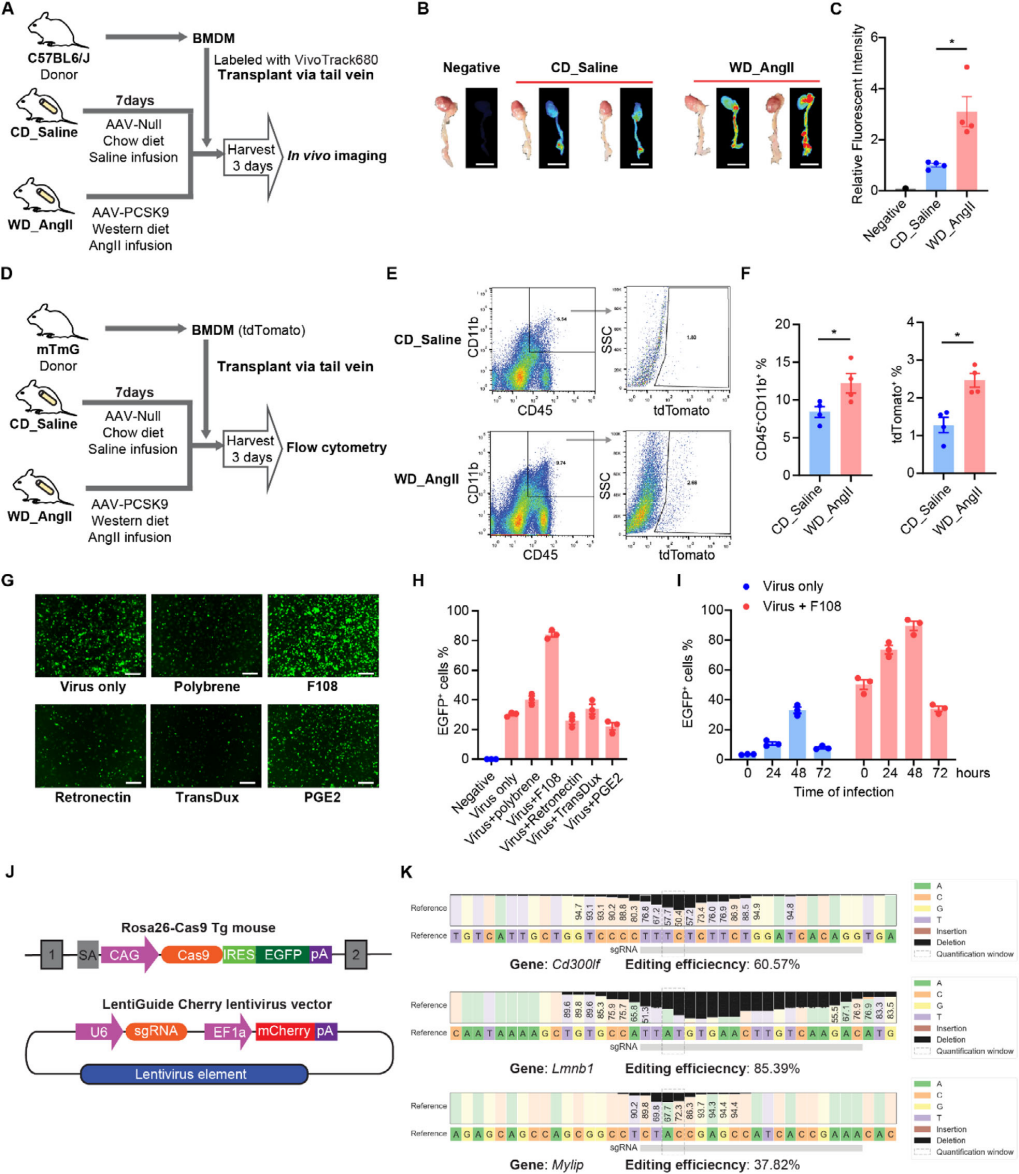
Development of in vivo genome‐wide CRISPR Cas9 screen for monocyte infiltration (CRISPR‐MI). A) Flowchart of the adoptive transfer of BMDM labeled with VivoTrack680 fluorescent dye. B,C) The aortae from recipient mice in (A) were isolated and underwent in vivo imaging (n = 4). Scale bar = 1 cm. D) Flowchart of the adoptive transfer of BMDM from mTmG mice. E,F) The aortae from recipient mice in (D) were digested and analyzed by flow cytometry (n = 4). G, BMDMs were infected with lentivirus encoding EGFP (lenti‐EGFP, 50 MOI) 48 h after isolation with different adjuncts. 22 h after infection, cells were imaged by fluorescent microscopy. Scale bar = 100 µm. H) BMDMs in (G) were analyzed by flow cytometry to quantify EGFP+ cells (n = 3). I) BMDMs, were infected with lenti‐EGFP at different time points (0, 24, 48, 72 h) after isolation with or without F108. EGFP^+^ cells were quantified by flow cytometry (n = 3). J) Rosa26‐Cas9 Tg mice were used as the BMDM donor, and the LentiGuide Cherry lentivirus vector was used to carry sgRNA. K) BMDMs from Rosa26‐Cas9 Tg mice were infected with lentivirus carrying different sgRNAs (50 MOI) 48 h after isolation. 72 h after infection, the genome editing efficiency was determined by CRISPR‐Seq. Data are presented as mean ± SEM. Unpaired t‐test was used for C, F. ^*^, p < 0.05. CD, chow diet; WD, Western diet; AngII, angiotensin II; Tg, transgenic.

To acquire enough monocytes for the screen, we used the adoptive transfer method to transplant in vitro differentiated monocytes back into mouse circulation via tail vein injection.^[^ ^44^ ^]^ Although macrophage adoptive transfer has been used to study the effect of educated macrophages on various disease development, including pulmonary fibrosis,^[^ ^44^ ^]^ cancer immunotherapy,^[^ ^45^ ^]^ and sepsis,^[^ ^46^ ^]^ it has not been well‐studied in aortic aneurysm models. We prepared the monocyte from the mouse bone marrow isolated from the mouse femur and tibia. Usually, isolated bone marrow was cultured in a medium containing murine macrophage colony‐stimulating factor (M‐CSF) for 7 days to facilitate its differentiation to macrophage.^[^ ^47^ ^]^ Based on previous publications^[^ ^48^, ^49^, ^50^ ^]^ and our results, bone marrow cells at day 5 showed higher Ly6C and lower F4/80 expression (Figure S1, Supporting Information). As a result, we chose to culture bone marrow cells for 5 days to maintain them in a monocyte‐like status instead of the mature macrophage status for better recapitulating the monocyte infiltration process in vivo. Using this method, we were able to acquire ≈2 × 10^7^ cells from every mouse. To test whether the bone marrow‐derived monocytes (BMDMs) are functional to infiltrate into the aorta, we first used fluorescent dye (VivoTrack680) to label the BMDMs before transplantation via tail vein injection (**Figure** **1**A). Three days after transplantation, we determined the fluorescence in the aorta from healthy control (AAV‐Null + chow diet + saline infusion) and AAA model (AAV‐PCSK9 + Western diet + AngII infusion). The aorta from the AAA model group showed a higher fluorescent signal, indicating more infiltrated monocytes (Figure 1B,C). Additionally, we used BMDMs from mT/mG mice (The Jackson Laboratory Strain 007676), which express cell‐membrane‐localized tdTomato (mT) in all cells/tissues (Figure 1D). After transplantation, mT‐positive cells were determined by flow cytometry. Consistently, the aorta from the AAA model group contained significantly more mT‐positive macrophages (Figure 1E,F).

Lentivirus is the most common tool for carrying sgRNA into cells in a CRISPR screen.^[^ ^51^, ^52^ ^]^ However, primary myeloid cells, including monocytes, macrophages, and dendritic cells, are difficult for lentivirus to infect.^[^ ^53^, ^54^ ^]^ To successfully perform the CRISPR screen in monocytes, we need to optimize the lentivirus infection protocol to be compatible with the current CRISPR screen platform. We infected BMDMs with lentivirus encoding EGFP (lenti‐EGFP) and examined the efficacy of several reported infection‐enhancing adjuvants in BMDMs, including polybrene,^[^ ^55^, ^56^ ^]^ Poloxamer synperonic F108 (F108, another name is P338),^[^ ^57^, ^58^, ^59^ ^]^ Retronectin,^[^ ^60^, ^61^ ^]^ TransDux,^[^ ^62^ ^]^ and Prostaglandin E2 (PGE2).^[^ ^57^, ^63^ ^]^ The fluorescent image and quantification by flow cytometry showed that only F108 could significantly increase the infection rate (84.10%), compared with the virus‐only group (30.26%) (Figure 1G,H). Interestingly, we found that the time of lentivirus supplementation is critical for the infection efficiency. If the lenti‐EGFP was added immediately after bone marrow isolation (0 h), the infection rate would be extremely low (3.5%) (Figure 1I). We determined the optimal time for adding lenti‐EGFP after bone marrow isolation was 48 h. Combined with optimal time point and F108 adjuvant, we were able to increase infection efficiency to 89.5% (Figure 1I). In this way, we could effectively infect BMDMs with lentivirus with minimal lentivirus dosage during the screen.

Although CRISPR‐Cas9‐mediated genome editing is highly efficient in cell lines, it is generally more challenging in primary cells. In the CRISPR screen, low genome editing efficiency could induce noise in the results and decrease the power to find true targets. To maximize the editing efficiency, we used transgenic mice (The Jackson Laboratory Strain 028555, Rosa26‐Cas9 mouse), which constitutively expressed Cas9 protein as BMDM donors (Figure 1J). After transducing BMDMs with lentivirus carrying different sgRNAs (Figure 1J), with the aforementioned protocol, the genome editing efficiency was determined by next‐generation sequencing (NGS). We randomly chose 3 gRNA sequences, and the results showed that the editing efficiency is 60.57% for *Cd300lf* gene, 85.39% for *Lmnb1* gene, and 37.82% for *Mylip* gene, respectively (Figure 1K). In summary, we established an optimized BMDM lentivirus infection and adoptive transfer method for CRISPR‐MI.

### 2.2 CRISPR‐MI Identified Causal Genes Regulating Monocyte Infiltration in Murine AAA Model

**Figure 2:**
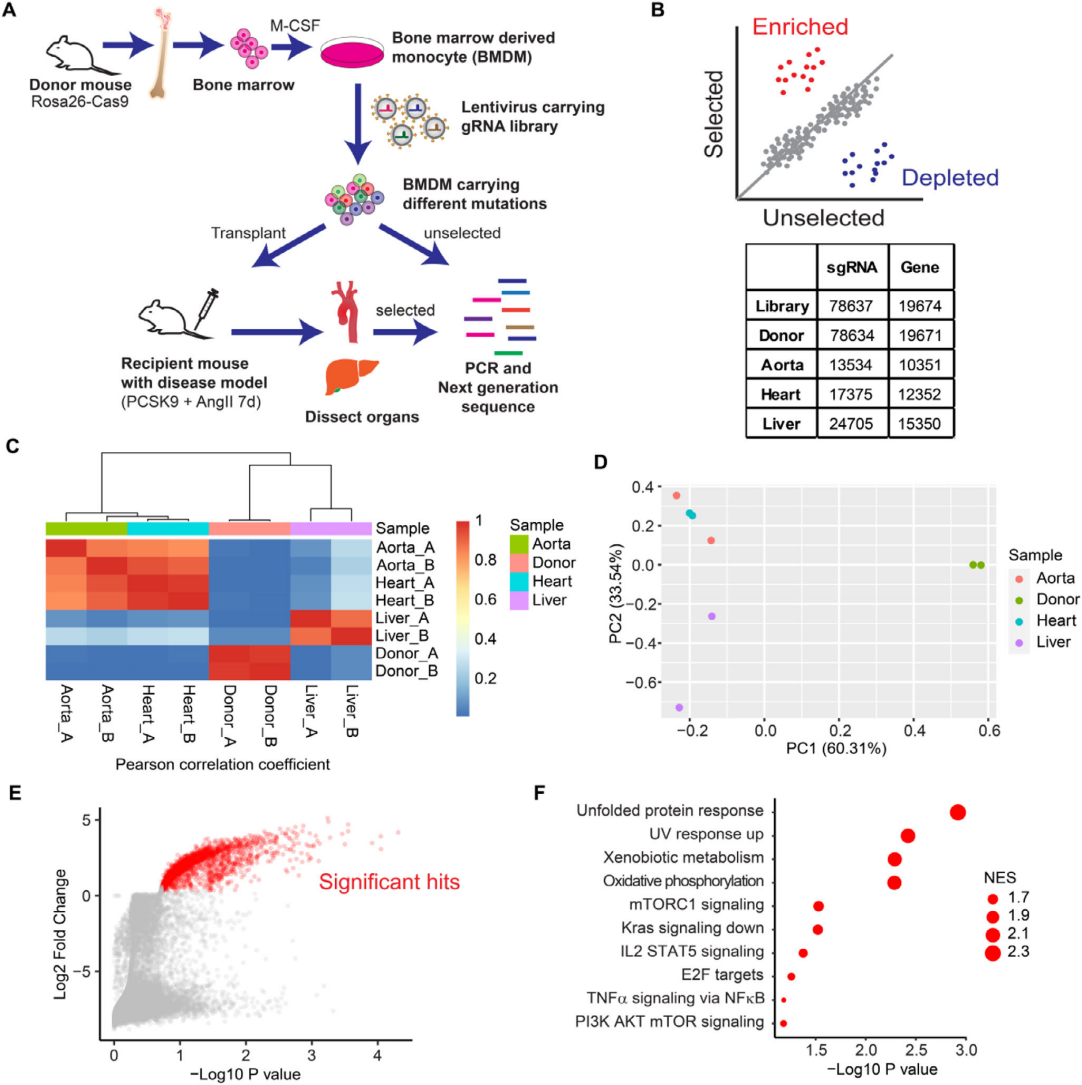
CRISPR‐MI identified genes regulating monocyte infiltration in the murine AAA model. A) Flowchart of CRISPR‐MI. B) Number of sgRNA and corresponding genes identified by next‐generation sequencing (NGS) in BMDM donor and aorta, heart, and liver from recipient mice. C) Heat plot showing the Pearson correlation coefficient of gRNA counts from different organs and biological replicates. D) Principal component analysis gRNA counts from different organs and biological replicates. E) Scatter plots comparing gRNA targeting genes in aorta versus gDNA in donor BMDMs. Red dots indicated significantly enriched genes in the aorta. F) Scatter plot of overrepresented pathways of genes enriched in the aorta by GSEA.

We designed the CRISPR‐MI workflow in **Figure** **2**A. The BMDMs were acquired from Rosa26‐Cas9 mice and differentiated in vitro. 48 h after bone marrow isolation, lentivirus‐carrying sgRNA library were added to BMDMs with F108. We used the genome‐wide sgRNA library (Mouse Cherry Brie Pooled Library, from Addgene Pooled Library #170511)^[^ ^64^ ^]^ to perform the genome‐wide CRISPR knockout screen. We chose this library because 1) The gRNAs were designed by improved computational rules with fewer off‐targets^[^ ^65^ ^]^; 2) It carries mCherry cassette for easy determination of infection rate. To avoid one cell carrying multiple sgRNAs, we optimized the lentivirus dosage (30MOI) and checked the infection rate to be ≈30–50% by mCherry expression. Five days after bone marrow isolation, 2 × 10^7^ BMDMs were transplanted into AAA model mice (AAV‐PCSK9 (21 days) + WD (21 days) + AngII (7 days)). Three days after transplantation, the mice were sacrificed, and the aorta, heart, and liver were harvested for PCR amplification and NGS for sgRNAs. Our hypothesis is that if one sgRNA was enriched in the aorta, it indicated that knockout of the corresponding gene promoted the monocyte infiltration into the aorta.

We used the MAGeCK method^[^ ^66^, ^67^ ^]^ to analyze the NGS results of sgRNA abundance in different organs, compared with sgRNA abundance in donor BMDMs before transplantation (as unselected background control). The original sgRNA library contains 78 637 gRNAs, corresponding to 19 674 mouse genes. In the donor BMDMs, we were able to recover 78 634 gRNAs and 19 671 genes (Figure 2B), indicating enough coverage of genes in the mouse genome. After adoptive transfer, we could detect 13 534 sgRNA (10 351 genes) in the aorta, 17 375 sgRNA (12 352 genes) in the heart, and 24 705 sgRNA (15 350 genes) in the liver. We did 2 biological replicates for each organ, and we calculated the Pearson correlation coefficient between different samples (Figure 2C). We also performed PCA analysis for each sample (Figure 2D). The high correlation of the repeats indicated the high repeatability of the CRISPR‐MI method. In the results, we found that sgRNA reads from the aorta and heart showed higher similarity compared to sgRNA reads from the liver. It indicated that monocyte infiltration into the aorta and heart may be regulated by similar genes or pathways. The screening results of aorta are shown in Figure 2E and Table S1 (Supporting Information). We select positive genes in our screen that at least 2 out of 4 sgRNAs reach significance (FDR P value < 0.05).^[^ ^68^ ^]^ The gene set enrichment analysis (GSEA) results of enriched gene sets in the aorta included unfolded protein response,^[^ ^69^ ^]^ oxidative phosphorylation,^[^ ^70^ ^]^ mTORC1 signaling,^[^ ^71^ ^]^ and TNFα signaling via NF‐κB,^[^ ^72^ ^]^ which are known to be implicated in monocyte infiltration or aortic aneurysm (Figure 2F). Importantly, to validate our CRISPR‐MI results, we used siRNA to knockdown the top hits (*Abhd6*, *Ascl4*, *Chrna9*, *Dusp28*, *Micu2*, *Gpr137b*, *Gpr37l1*, *Sp6*, *Marveld1*, *Tmem178*, and *Znrf1*) in Raw264.7 cell line and determined the expression of genes involved in inflammation (*Tnf*, *Il1b*, *Il6*, and *Il10*) and M1 macrophage polarization (*Cd86*, *Cd80*, and *Nos2*). Ten out of eleven genes, except for *Marveld1*, increased at least one gene in inflammation or M1 macrophage polarization (Figure S2, Supporting Information).

### 2.3 CRISPR‐MI and scRNA‐Seq Identified TREM2 as a Likely Critical Regulator of Monocyte Infiltration in AAA

**Figure 3:**
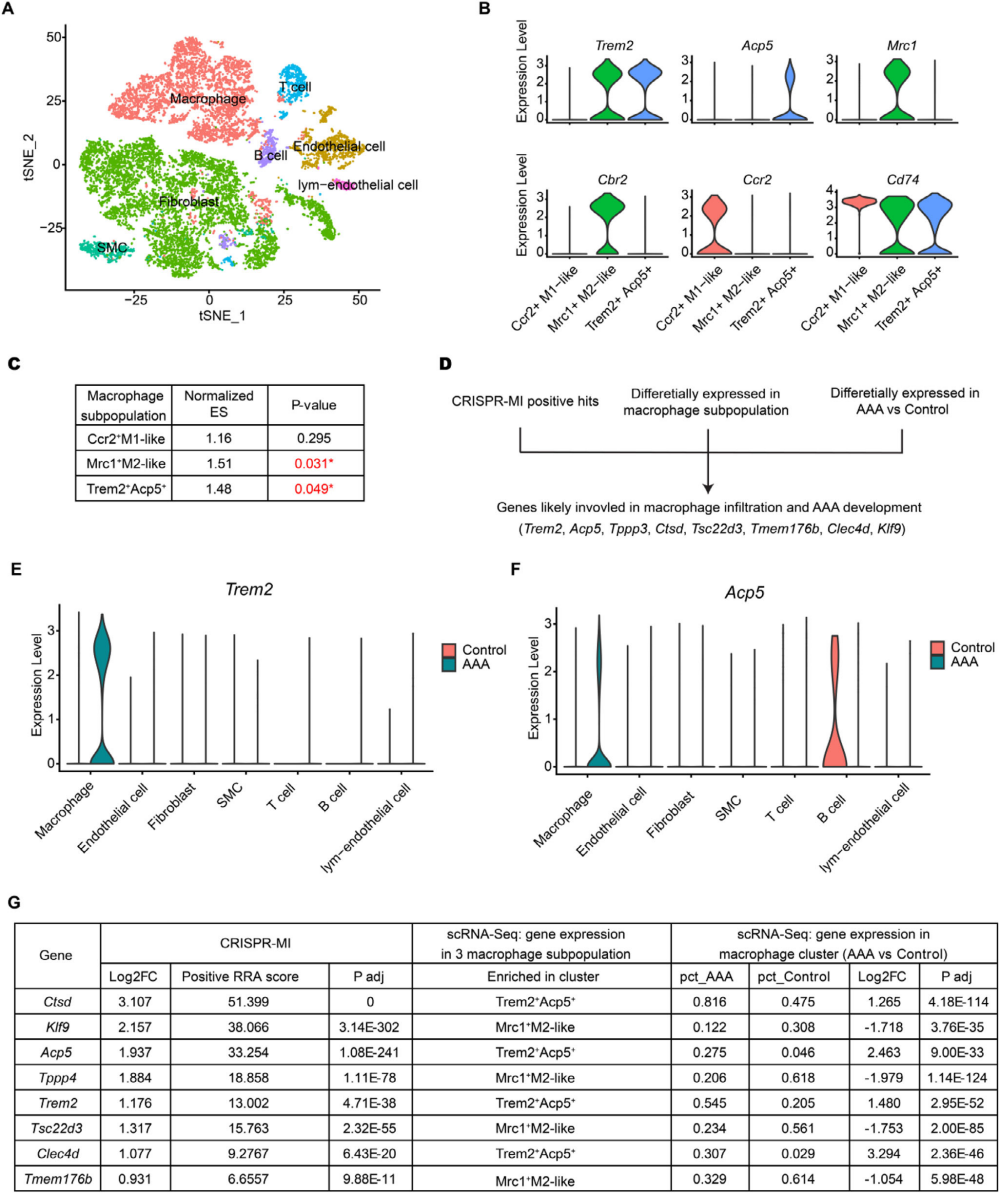
CRISPR‐MI and scRNA‐Seq identified TREM2 as a likely critical regulator of monocyte infiltration in AAA. A) T‐SNE representation of the aligned gene expression data from saline‐ (Control) and AngII‐ (AAA) treated ApoE mouse aortae. B) Violin plot showing differentially expressed genes in 3 macrophage subpopulations. C) GSEA result of enriched genes in CRISPR‐MI, using marker genes of each macrophage subpopulation as a gene set. D) Criteria for finding genes likely involved in macrophage infiltration and AAA development. E,F) Violin plot showing *Trem2* and *Acp5* expression in each cluster. G) Information of genes identified in D. ^*^, p <0.05. Log2FC, log2 fold change; P adj, adjusted p‐value; pct, percentage of cells expressing the gene.

To ensure the physiological and pathological relevance of the CRISPR‐MI positive hits in AAA development, we further leveraged the scRNA‐Seq data of the mouse AAA model to refine the 2516 genes with false discovery rate (FDR)<0.05 in the aorta from CRISPR‐MI (**Figure** **3**A).^[^ ^43^ ^]^ In the scRNA‐Seq dataset, monocytes/macrophages in mouse aorta could be classified into three subgroups: *Ccr2* ^+^ M1‐like, *Mrc1* ^+^ M2‐like, and *Trem2* ^+^/*Acp5* ^+^ (Figure 3B). Among them, *Ccr2* ^+^ M1‐like macrophages express a higher level of pro‐inflammatory cytokines, and *Mrc1* ^+^ M2‐like macrophages are supposed to be anti‐inflammatory and involved in tissue repair.^[^ ^43^, ^73^ ^]^ The role of *Trem2* ^+^/*Acp5* ^+^ macrophage subpopulation is less clear. We used GESA to compare positive hits in our screen with marker gene lists of three distinct macrophage clusters in scRNA‐Seq. We found that the genes identified as significantly enriched in our screen were enriched in the marker gene list of *Mrc1* ^+^ M2‐like and *Trem2* ^+^/*Acp5* ^+^ macrophage clusters (Figure 3C). These findings suggested that the positive genes from our screen putatively have an anti‐infiltrating or anti‐inflammatory role in the AAA model. The GSEA results further indicated a possibility that *Mrc1* ^+^ M2‐like and *Trem2* ^+^/*Acp5* ^+^ macrophages are both anti‐inflammatory. We refined our gene list by analyzing the genes met three key requirements: 1) genes identified as having a direct causal role in monocyte infiltration from our CRISPR‐MI screen; 2) genes differentially expressed across various macrophage subpopulations, highlighting the functional diversity that may exert protective or harmful effects in disease states; and (3) genes that are highly and differentially expressed in macrophages in AAA compared to control conditions, indicating significant disease relevance (Figure 3D). In total, 8 genes were identified as genes most likely to be involved in macrophage infiltration and AAA development, including *Trem2*, *Acp5*, *Tppp3*, *Ctsd*, *Tsc22d3*, *Tmem176b*, *Clec4d*, and *Klf9*.

Interestingly, *Trem2* and *Acp5*, both marker genes of the *Trem2* ^+^/*Acp5* ^+^ macrophage cluster, were identified as positive hits in our screen. We further analyzed the expression of these genes in the AAA scRNA‐seq dataset. *Trem2* was most abundant in macrophage clusters and was highly inducible after AngII induction in the AAA model (Figure 3E). Immunofluorescence staining also demonstrated that TREM2 was predominantly expressed in macrophage (Figure S3A, Supporting Information). *Acp5* had a similar pattern, but it is also expressed in B cells from the control mouse aorta (Figure 3F). *Clec4d* was also induced in the AAA model (Figure S3B, Supporting Information). The rest of the genes were expressed in a wide range of cell types (Figure S3B, Supporting Information). Detailed information of these eight genes is shown in Figure 3G.

Combining the screen results and the single‐cell RNA‐seq data, we hypothesized that *Trem2* could be an important regulatory gene of monocyte infiltration in AAA. Given its significant implications, *Trem2* has been selected for further in‐depth biological function studies.

### 2.4 *Trem2* KO Promoted BMDM Adhesion, Migration, and Infiltration into the Aorta Following AngII Infusion

**Figure 4:**
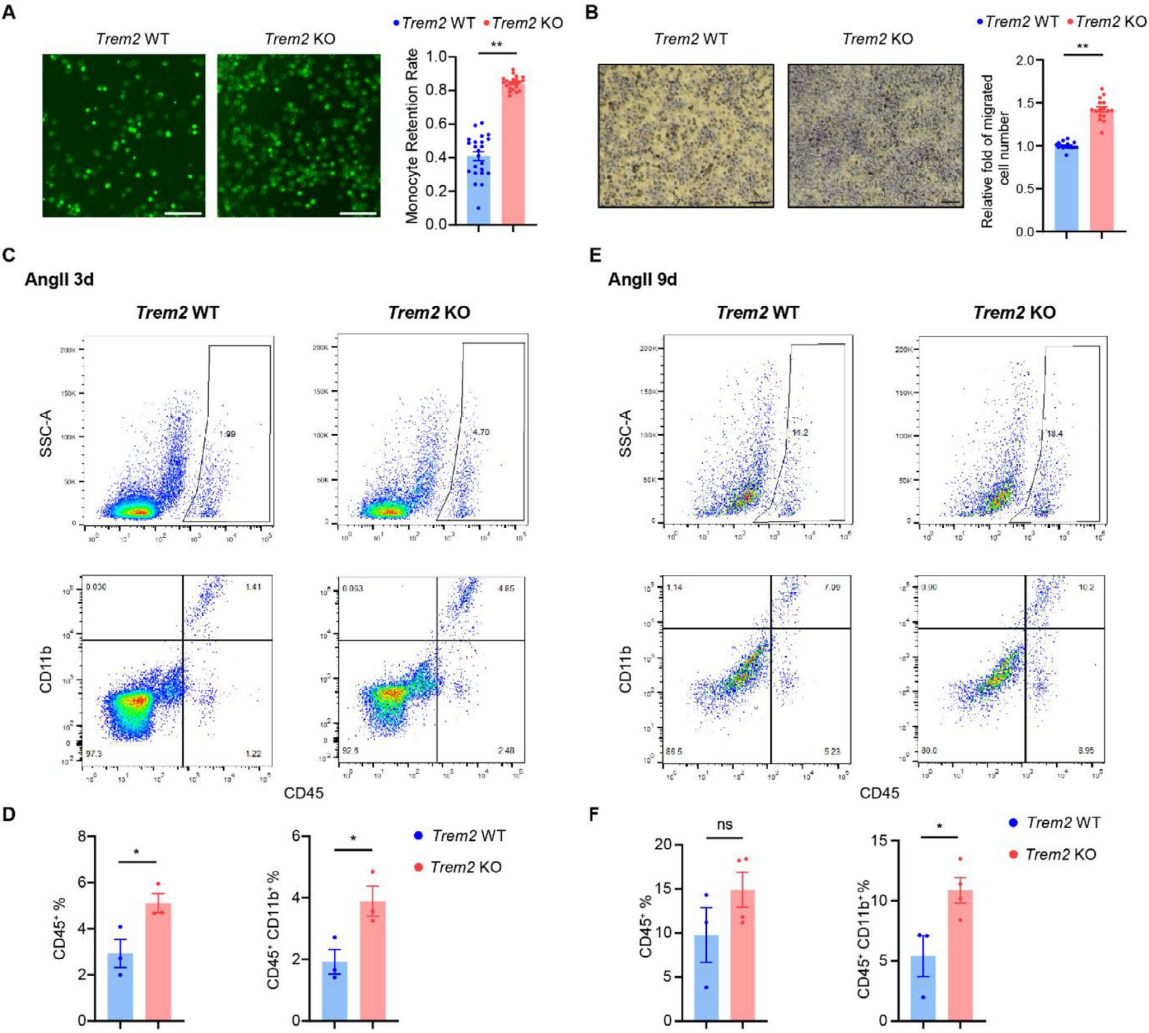
*Trem2* KO promoted BMDM adhesion, migration, and infiltration into the aorta following AngII infusion. A) BMDMs from *Trem2* WT and KO mice were stained with Calcein AM, and BMDMs attached to mouse aortic endothelial cells were imaged or quantified by plate reader (n = 22). Scale bar = 100 µm. B) Boyden chamber assay was utilized to assess the transwell migration of BMDMs from *Trem2* WT and KO mice. Migrated BMDMs were stained with Crystal Violet and quantified under microscopy (n = 16). Scale bar = 200 µm. C–F) Mice were injected with AAV‐PCSK9 and fed a Western diet for 2 weeks, followed by AngII (1500 ng kg^−1^ min^−1^) infusion for 3 days (C‐E, n = 3) or 9 days (D–F, n = 3 to 4). The aortae were digested and stained for cytometry analysis. CD45^+^ indicates all immune cells. CD45^+^/CD11b^+^ indicates macrophage. To avoid the contamination of blood, aortas with dissection were excluded from the analysis. Data are presented as mean ± SEM. Unpaired t‐test was used for A, B, D, F. ^*^, p <0.05; ^**^, p <0.01.

To elucidate the role of *Trem2* in monocyte and macrophage function, we first conducted in vitro assays using BMDMs from *Trem2* KO and WT mice. Adhesion assays demonstrated that *Trem2* KO BMDMs, post‐PBS flushing, had a heightened capacity to retain on mouse aortic endothelial cells, indicating increased adhesion (**Figure** **4**A). Additionally, we evaluated the migration capacity of BMDMs toward cultured mouse primary aortic smooth muscle cells using the Boyden chamber assay, which revealed that *Trem2* KO significantly enhanced the migration (Figure 4B).

To further assess the implications of *Trem2* KO in vivo, we generated hypercholesterolemia and AngII induced AAA in *Trem2* KO and WT mice. *Trem2* KO was validated by Western blot (Figure S4, Supporting Information). The immune cell population in the aorta was analyzed by flow cytometry. Both CD45^+^ (pan immune cells) and CD45^+^/CD11b^+^ cells (macrophages) are increased in the aorta from *Trem2* KO mice, compared to WT mice, after 3 days of AngII infusion (Figure 4C,D). After 9 days of AngII infusion, CD45^+^/CD11b^+^ cells are still significantly increased in the aorta from *Trem2* KO mice. The CD45^+^ cells did not show a significant difference, possibly due to increased infiltration of other immune cells (dendritic cells, mast cells, and lymphocytes^[^ ^74^ ^]^), which diluted the effect of macrophage number difference (Figure 4E,F). These results demonstrated that *Trem2* KO indeed promoted early monocyte infiltration into the aorta in the mouse AAA model, aligning with our CRISPR‐MI screen results.

### 2.5 Trem2 KO Enhances Expression of Adhesion and Inflammatory Genes in BMDMs Partially Through ERK Activation

**Figure 5:**
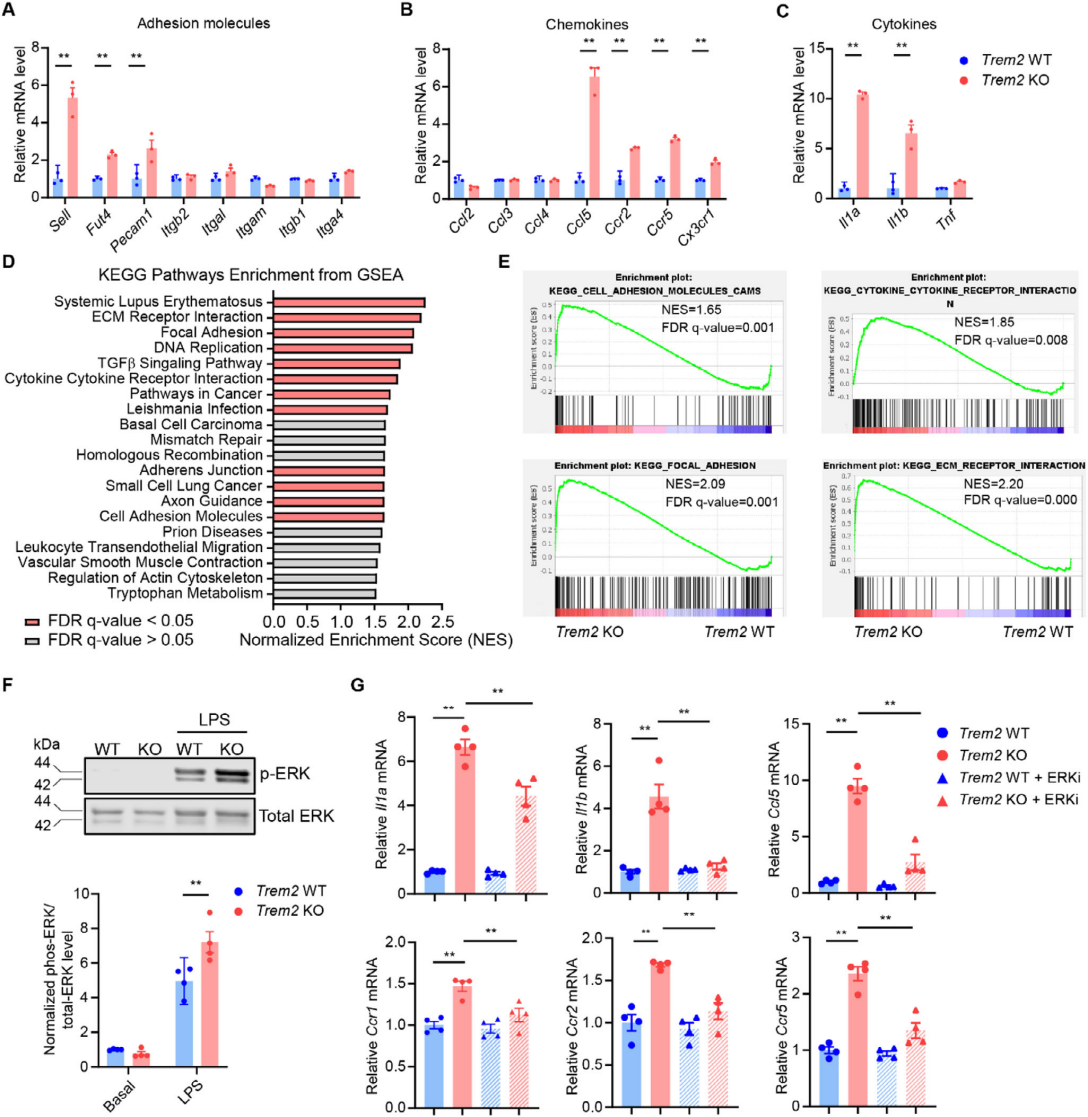
*Trem2* KO enhances expression of adhesion and inflammatory genes in BMDMs partially through ERK activation. A–C) Expression of genes involved in adhesion (A), chemotaxis (B), and cytokines (C) in BMDMs from *Trem2* WT and KO mice was quantified by qPCR (n = 3). D) Transcriptomes of BMDMs from *Trem2* WT and KO mice were assessed by RNA‐Seq. Bar chart showing KEGG pathways enriched in *Trem2* KO BMDMs. E) Enrichment plot of KEGG pathways, including cell adhesion molecules, cytokine‐cytokine receptor interactions, focal adhesion, ECM receptor interaction. F) BMDMs were treated with/without LPS (1 µg mL^−1^) for 1 h and harvested for Western blot (n = 4). G) BMDMs from *Trem2* WT and KO mice were treated with ERK inhibitor (ERKi, GDC‐0994, 50 nM) for 6 h and harvested for qPCR (n = 4). Data are presented as mean ± SEM. Two‐way ANOVA was used for A, B, C, F, G. ^*^, p <0.05; ^**^, p <0.01.

Quantitative PCR (qPCR) analysis showed that *Trem2* KO significantly increased the expression of several adhesion molecules and enzymes responsible for adhesion molecule synthesis (*Sell*, *Fut4*, *Pecam1*), chemotactic molecules (*Ccl5*, *Ccr2*, *Ccr5*, *Cx3cr1*), and cytokines (*Il1a*, *Ilb)* in BMDM (**Figure** **5**A–C). Chemotactic molecules and cytokines secreted into the medium were determined by ELISA, and the result confirmed that *Trem2* KO BMDM released significantly more IL6, IL12p40, TNFα, CCL2, CCL3, CCL4, VEGF, and PDGF‐BB (Figure S5A, Supporting Information). Moreover, to exclude that persistent *Trem2* KO could interfere with the BMDM differentiation and induced compensation mechanism, we also used siRNA to transiently knock down *Trem2* expression in BMDM at day 5. The qPCR results also demonstrated *Trem2* knockdown increased the expression of adhesion molecules and enzymes responsible for adhesion molecule synthesis (*Sell*, *Fut4*, *Pecam1*, *Itgb2*, *Itgal*, *Itgam*, and *Itga4*), chemotactic molecules (*Ccl5*, *Ccr2*, *Ccr5*, *Cx3cr1*), and cytokines (*Il1a*, *Il1b*) (Figure S6, Supporting Information).

To elucidate the molecular mechanism of *Trem2* in the cells, we compared the transcriptome of BMDMs from *Trem2* KO and WT mice (Table S2, Supporting Information). The GSEA result of the RNA‐Seq showed that pathways including ECM receptor interaction, focal adhesion, and cytokine‐cytokine receptor interaction are significantly overrepresented in the *Trem2* KO group (Figure 5D,E). The extracellular signal‐regulated kinase (ERK) pathway is important for macrophage inflammation and aortic aneurysm development.^[^ ^75^ ^]^ *Trem2* KO significantly increased phosphorylation of ERK (Figure 5F). ERK inhibitor (GDC‐0994) diminished or abolished the effect of *Trem2* KO on *Il1a*, *Il1b*, *Ccl5*, *Ccr1*, *Ccr2*, *Ccr5* expression in BMDM (Figure 5G). In addition, we also assessed other important inflammatory pathways in BMDM, including NF‐κB, PI3K, and AKT pathways, but we did not observe a significant change in these pathways between WT and *Trem2* KO BMDM (Figure S5B, Supporting Information). In short, we found that *Trem2* KO enhanced the adhesion and migration ability of BMDMs consistently with CRISPR‐MI results. This phenotype was at least partially mediated by increased ERK pathway activity.

### 2.6 TREM2 Levels Correlate with Human AAA and its Deficiency Protects Against AAA in Mice

**Figure 6:**
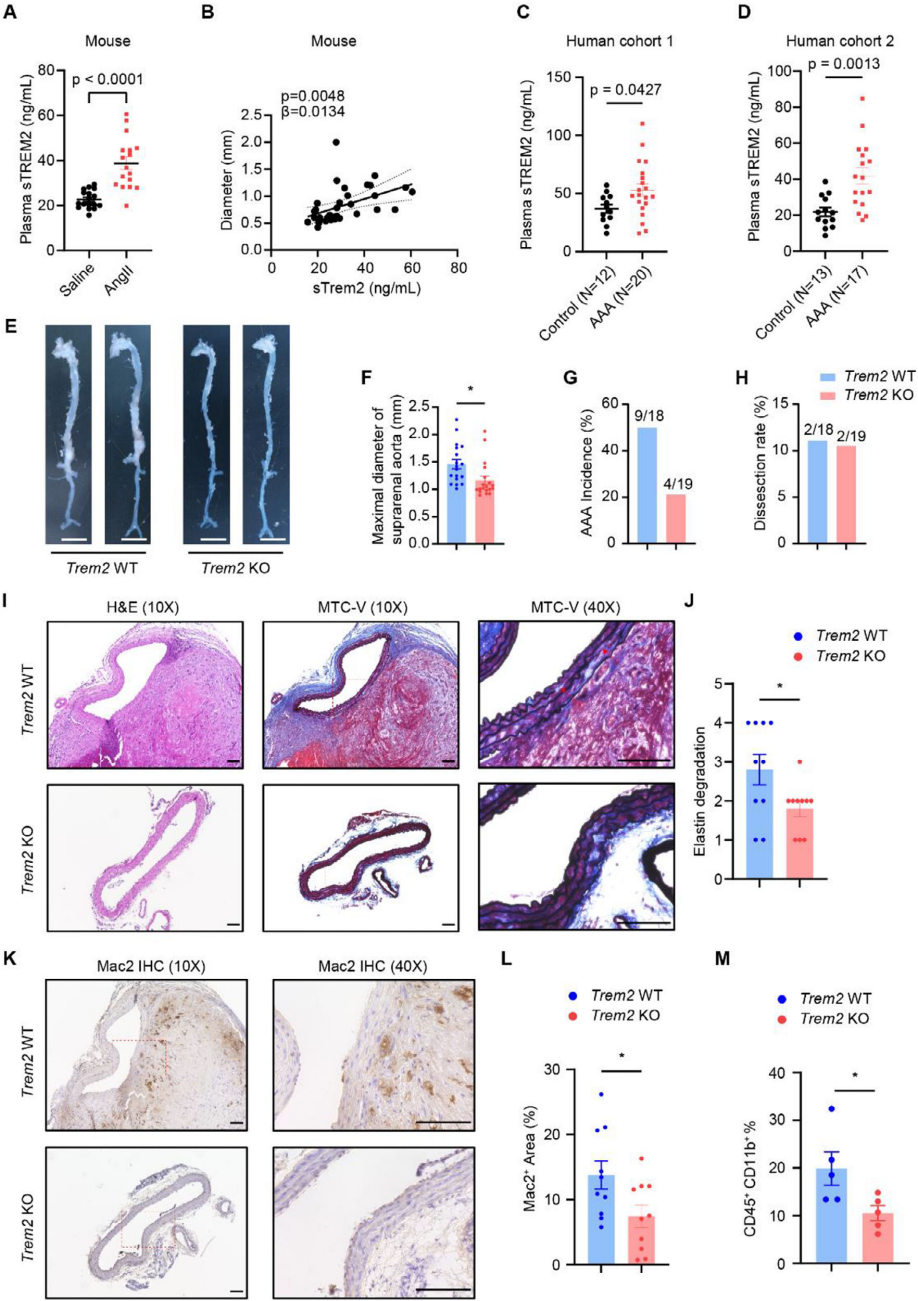
TREM2 levels correlate with human AAA, and its deficiency protects against AAA in mice. A) Mice were injected with AAV‐PCSK9 and fed with a Western diet for 2 weeks, followed by saline or AngII (1500 ng kg^−1^ min^−1^) infusion for 4 weeks. Plasma soluble TREM2 (sTREM2) was determined by ELISA (n = 14‐18). B) The scatter plot showing the relationship between maximal suprarenal aortic diameter and plasma sTREM2 in (A) (n = 32). C,D) sTREM2 levels in plasma from AAA patients and corresponding controls were determined by ELISA two human cohorts (C,D). E–L) *Trem2 WT and KO* mice were injected with AAV‐PCSK9 and fed with a Western diet for 2 weeks, followed by saline or AngII infusion for 4 weeks. The aorta (E) was examined for the maximal diameter of the suprarenal aorta (F, n = 18‐19), AAA incidence (G), and dissection rate (H). Scale bar = 0.5 cm. Hematoxylin and Eosin (H&E) staining and Masson trichome with a Verhoeff counterstaining (MTC‐V) (I) were used to determine the elastin degradation (J, n = 10). Macrophage infiltration was determined by immunohistochemistry (IHC) using Mac2 antibody (K,L, n = 10) and flow cytometry (M, n = 5). Scale bar = 100 µm. Data are presented as mean ± SEM. Unpaired t‐test was used for (A–D,F,J,L,M). ^*^, p <0.05. The arrow indicates the elastin break.

To explore the role of TREM2 in the AAA, we first analyzed soluble TREM2 (sTREM2) in plasma from mice infused with saline or AngII after AAV‐PCSK9 injection and Western diet (WD) feed. sTREM2 level was significantly higher in the AngII group (**Figure** **6**A). We also analyzed plasma samples of AAA patients and corresponding controls from 2 different medical centers. sTREM2 level was determined by ELISA, and both cohorts demonstrated that sTREM2 is significantly higher in AAA patients (Figure 6C,D).

Further insights were gained from RNA sequencing of human AAA lesions collected during surgery. We performed weighted correlation network analysis (WGCNA)^[^ ^76^ ^]^ for the top 10 000 most variant genes (DESeq2 normalized count > 10) extracted from these 17 human patient RNA‐Seq data, resulting in a total of 21 co‐expression modules (power > 0.9) (Figure S7A,B, Supporting Information). *TREM2* emerged as a hub gene within the “yellow” module (Figure S7C, Supporting Information), which was significantly enriched in pathways related to leukocyte extravasation and inflammation (Figure S7D, Supporting Information). This suggests that TREM2 may be a key driving factor in modulating leukocyte dynamics and inflammatory responses in human AAA, with variations in *TREM2* expression likely reflecting different disease stages. Indeed, we have found the plasma sTREM2 levels correlate positively with aortic diameter in AAA mice (Figure 6B).

To investigate the functional role of TREM2 in vivo, we used *Trem2* KO and WT mice for the AAA model (PCSK9+AngII). In this model, *Trem2* KO did not influence plasma total cholesterol, plasma total triglyceride, systolic blood pressure, or body weight (Figure S8, Supporting Information). *Trem2* KO did not affect white blood cell count (WBC) or WBC differential count (Figure S9A–C, Supporting Information). We used flow cytometry to further dissect the monocyte phenotype in *Trem2* KO and WT mice. We found that *Trem2* KO increased CD62L^+^/Ly6C^high^ monocyte percentage in blood in both basal (Figure S9D,E, Supporting Information) and AngII infusion (Figure S9F,G, Supporting Information) conditions. To further confirm that *Trem2* KO promoted the monocyte to become more pro‐inflammatory, we isolate the peripheral blood mononuclear cells (PBMCs) from *Trem2* KO and WT mice by density gradient centrifugation^[^ ^77^ ^]^ and quantified the expression of adhesion molecules, chemotactic molecules, and cytokines. Similar to the BMDM results, *Trem2* KO increased the expression of *Sell*, *Pecam1*, *Itgb2*, *Itgam*, *Itgb1*, *Itga4*, *Ccl5*, *Ccr5*, *Il1b*, and *Tnf* (Figure S9H–J, Supporting Information) in PBMCs.

Surprisingly, we found that *Trem2* KO attenuated the AAA development *in* the mouse model (Figure 6E). *Trem2* KO decreased the maximal suprarenal aorta diameter and AAA incidence but not the dissection rate (Figure 6F–H). In histology, we also determined that the aorta from the *Trem2* KO mouse showed less elastin degradation (Figure 6I,J) and macrophage number (Figure 6K–M). In female mice, *Trem2* KO did not change plasma cholesterol and showed a trend to decrease maximal suprarenal aorta diameter (Figure S10, Supporting Information). The statistics were not significant, possibly due to the low incidence of AAA in WT female mice.

### 2.7 *Trem2* KO Promoted Macrophage Apoptosis in Vasculature

**Figure 7:**
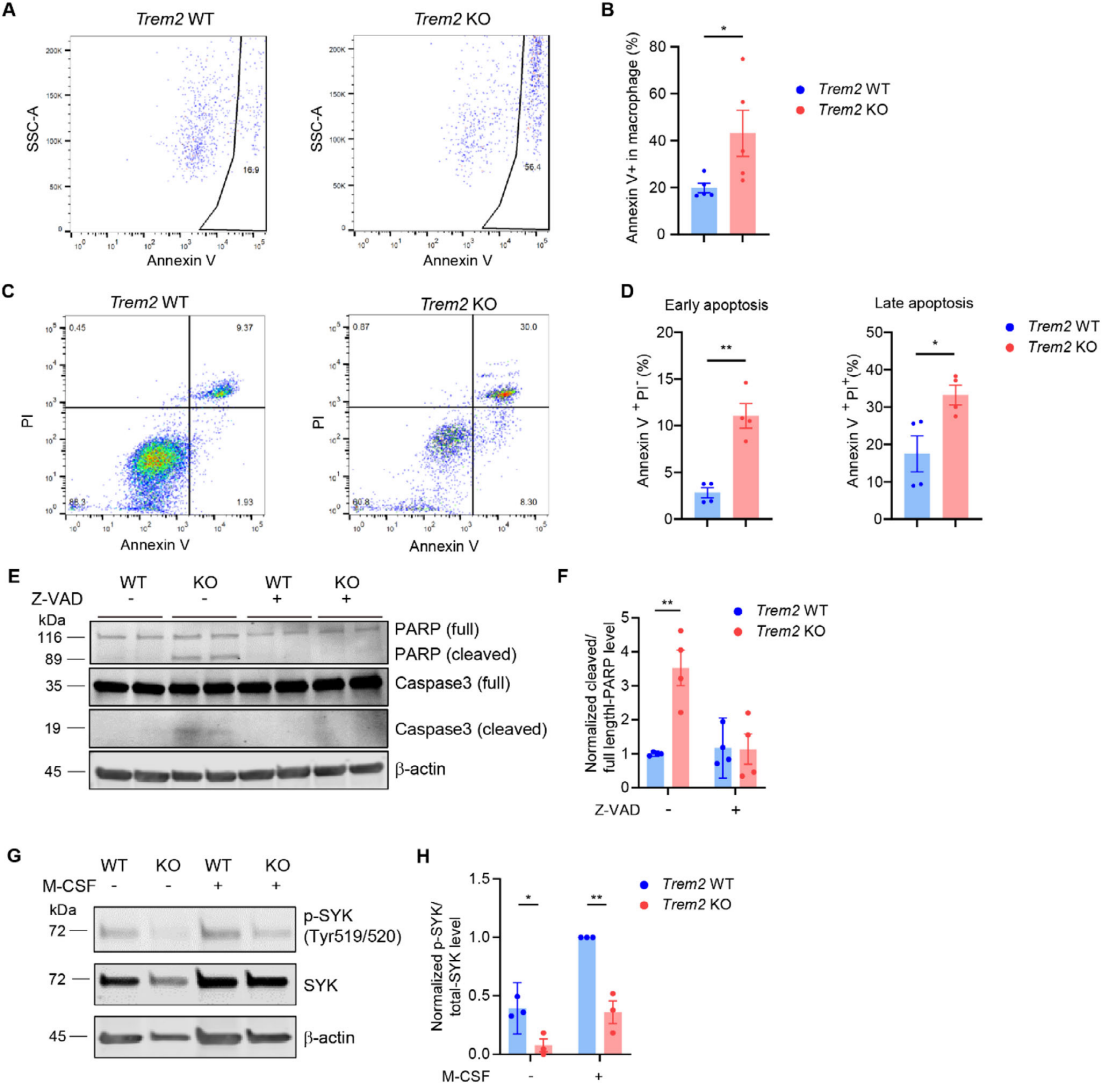
*Trem2* KO promoted macrophage apoptosis. A,B) *Trem2* WT and KO mice were injected with AAV‐PCSK9 and fed with a Western diet for 2 weeks, followed by saline or AngII infusion (1500 ng kg^−1^ min^−1^) for 4 weeks. The aorta was digested and stained with CD45, CD11b, and Annexin V‐FITC. Apoptotic macrophages were determined as CD45^+^/CD11b^+^/Annexin V^+^ cells in the aorta (B, n = 5). C–F) BMDMs from *Trem2* WT and KO mice were cultured for 9 days in vitro, apoptotic cells were determined by PI/Annexin V staining (C,D), and Western blot for cleavage of PARP1 and Caspase3 (E‐F) (n = 4). The cleaved Caspase 3 band was too weak to be quantified. G,H) BMDMs from *Trem2* WT and KO mice were cultured with/without M‐CSF (30 ng mL^−1^) for 24 h and harvested for Western blot (n = 3). Data are presented as mean ± SEM. Unpaired t‐test was used for B, D. Two‐way ANOVA was used for F, H. ^*^, p <0.05; ^**^, p <0.01. Z‐VAD, Z‐VAD‐FMK, a pan caspase inhibitor; M‐CSF, macrophage colony‐stimulating factor; SYK, spleen tyrosine kinase.

The paradox of the macrophage number difference in the early (Figure 4) and advanced stages (Figure 6) of AAA development inspired us to investigate the fate of macrophage in the aorta from the *Trem2* KO mice. We determined the apoptotic macrophage in the aorta from *Trem2* KO and WT mice after AngII infusion by Annexin V staining. The apoptotic macrophage was significantly increased in *Trem2* KO mice on day 9 (Figure S11A, Supporting Information) and 28 (**Figure** **7**A,B). Immunofluorescence staining also showed that *Trem2* KO increased macrophage apoptosis (MRC1 used as macrophage marker) on day 28 (Figure S11B, Supporting Information). In BMDM culture, we also found that both early and late apoptotic cells are increased after 7‐day culture in vitro (Figure 7C,D). *Trem2* KO also increased the cleavage of PARP and Caspase3, 2 indicators of apoptosis (Figure 7E,F). These data indicated that TREM2 is critical for macrophage survival.

The decreased survival of *Trem2 KO* BMDM was similar to the phenotype of DAP12 KO BMDM. DAP12‐deficient BMDM began to die after a 5‐day culture in vitro.^[^ ^78^ ^]^ DAP12 and TREM2 could form complexes to facilitate the biological function of CSF1R.^[^ ^79^ ^]^ M‐CSF/CSF1R is the most important signaling pathway for monocyte survival and differentiation. Notably, in the absence of M‐CSF, *Trem2* KO BMDMs exhibited increased apoptosis,^[^ ^80^ ^]^ though CSF1R expression was actually higher in these cells (Figure S12A,B, Supporting Information). In Western blot, we found that *Trem2* KO almost abolished the phosphorylation of spleen tyrosine kinase (SYK) (Tyr 519/520), an important downstream molecule of the M‐CSF receptor (Figure 7G,H), with or without M‐CSF. Under microscope, it is clear that *Trem2* KO induces massive loss of live cells, and this phenotype could be rescued by excessive M‐CSF (Figure S12C, Supporting Information). We also determined the autophagic pathways in *Trem2* WT and KO BMDMs, because extensive autophagy will also induce cell death. In Figure S12D (Supporting Information), autophagic pathways did not show significant change. Furthermore, we prepared the primary peritoneal macrophages (PMs) from *Trem2* WT and KO mice. Consistent with BMDMs, *Trem2* KO also induced massive loss of PMs during in vitro culture, which could be rescued by excessive M‐CSF (Figure S12E, Supporting Information). In short, we found that *Trem2* KO inhibited AAA development, likely due to decreased survival of macrophages via diminished CSF1R downstream pathway.

### 2.8 sTREM2 Overexpression Promoted AAA Development in Mice

**Figure 8:**
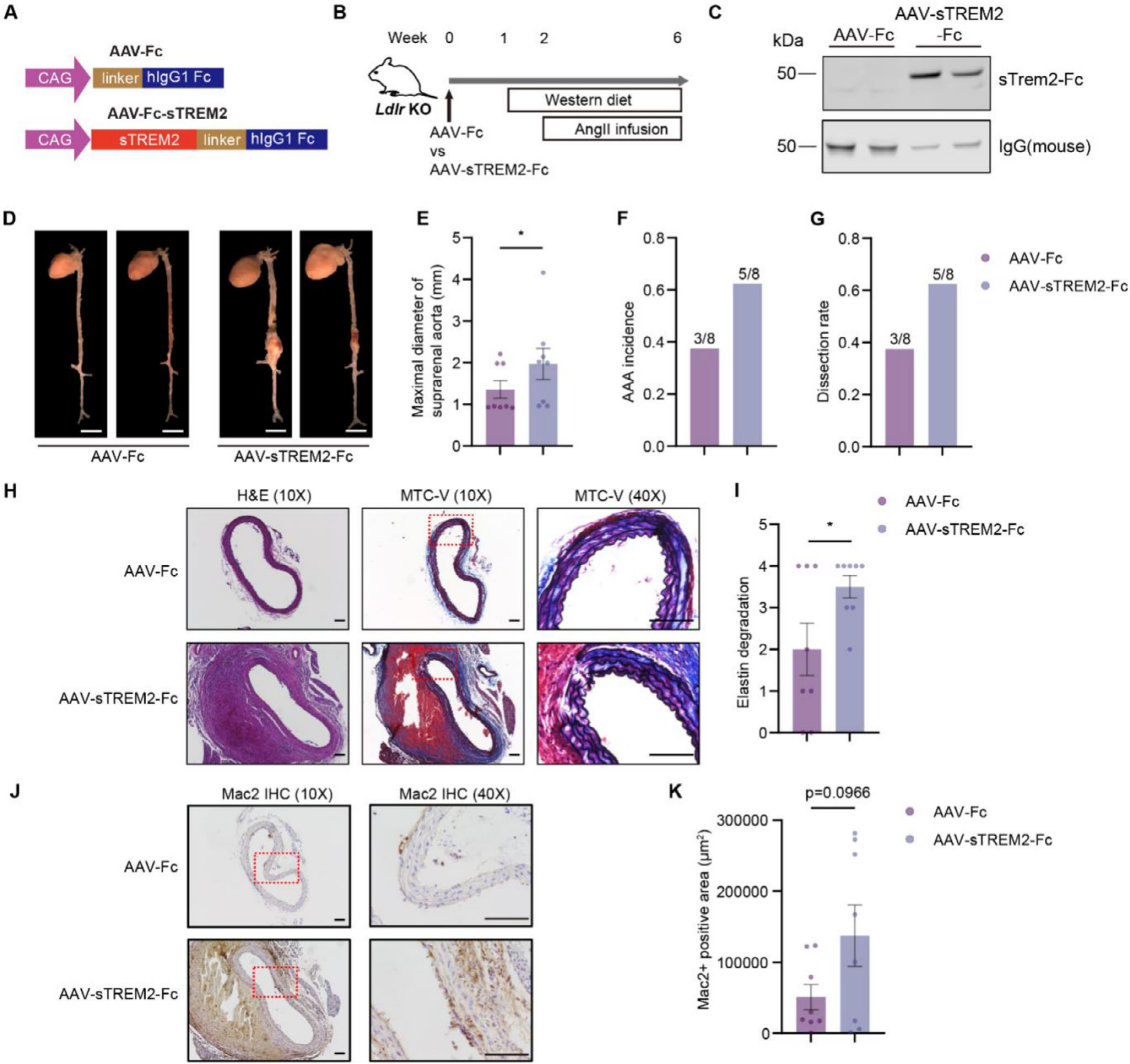
sTREM2 promotes AAA development in *Ldlr* KO mice. A) Schematics showing AAV vector for sTREM2‐Fc chimeric protein overexpression. B) Flowchart of sTREM2 overexpression in mouse AAA models. Six‐week‐old male *Ldlr* KO mice were injected with AAV‐Fc or AAV‐TREM2 and fed with Western diet 1 week ahead of AngII infusion, followed by AngII (1000 ng kg^−1^ min^−1^) infusion to induce AAA. C) Mouse plasma was used for Western blot. Mouse IgG was used as an internal control. D–K) The aorta (D) was examined for the maximal diameter of the suprarenal aorta (E), AAA incidence (F), and dissection rate (G). Scale bar = 0.5 cm. H&E and MTC‐V staining (H) were used to determine the elastin degradation (I). Macrophage infiltration was determined by immunohistochemistry (IHC) using Mac2 antibody (J,K) (n = 8). Scale bar = 100 µm. Data are presented as mean ± SEM. Mann‐Whitney test was used for E. Unpaired t‐test for I. Welch's *t*‐test for K. ^*^, p < 0.05.

Considering the secretory nature of TREM2 and its elevated levels in plasma from both human and mouse AAA samples, we investigated whether increased sTREM2 influences AAA development. Recombinant sTREM2 protein injection or adeno‐associated virus (AAV)‐mediated expression of sTREM2 has shown a protective role in Alzheimer's disease model,^[^ ^81^ ^]^ but its impact on AAA remains uncertain. TREM2 is cleaved by ADAM10/17 between His157 and Ser158 and released from the cell membrane.^[^ ^82^ ^]^ To further investigate the role of sTREM2 in AAA, we utilized AAV8 to overexpress sTREM2 in mouse AAA model. The sTREM2 (Met1‐His157) was fused with a human IgG1 Fc fragment to increase its secretion and half‐life in circulation (**Figure** **8**A).^[^ ^83^ ^]^ We established the AAA model by AngII infusion into *Ldlr* KO mice with the injection of AAV‐Fc or AAV‐sTREM2‐Fc (Figure 8B). After injection, the sTREM2‐Fc chimeric protein could be successfully detected in mouse plasma (Figure 8C). In this model, sTREM2 overexpression increased the maximal suprarenal aortic diameter, AAA incidence, and dissection rate (Figure 8D–G) without affecting plasma total cholesterol, triglyceride, or systolic blood pressure (Figure S13, Supporting Information). Opposite to the *Trem2* KO, sTREM2 overexpression increased elastin degradation (Figure 8H,I) and had a trend to increase macrophage number in the aortic wall (Figure 8J,K), potentially due in part to enhanced macrophage survival.^[^ ^80^, ^84^ ^]^ The results indicated that sTREM2 plays a detrimental role in AAA development.

## 3 Discussion

Macrophage is a key player in various cardiovascular diseases, including abdominal aortic aneurysms, thoracic aortic aneurysms, and atherosclerosis. The infiltration of monocytes into the aortic wall occurs in the early stages of aneurysm and atherosclerosis development. Targeting monocyte infiltration and activation could reduce tissue inflammation and remodeling. After AngII infusion in mice, the increase of macrophage in the aortic wall was primarily driven by infiltration of circulating monocytes in the early phase (< 10 days),^[^ ^14^ ^]^ suggesting that intervention of this response could reduce aortic inflammation and attenuate disease development. The recruitment of monocytes is known to be CCR2‐dependent,^[^ ^14^ ^]^ but the systematic study of genes regulating this process is still lacking. Our CRISPR‐MI method is a new tool to understand this complicated process. We optimized the lentivirus infection protocol and adoptive transfer method to facilitate the genome‐wide CRISPR screen in vivo and in disease conditions. By using this method, we identified novel regulators of monocyte infiltration.

Recently, scRNA‐Seq has provided insight into different macrophage clusters and differentially expressed genes in disease. However, scRNA‐Seq has a major limitation in that it cannot reveal causality between gene expression and the phenotype. Instead, our CRISPR‐MI began with functional screening and could infer the causal genes in monocyte infiltration. Meanwhile, the large number of positive hits from the CRISPR‐MI screen, while indicating a potential causal contribution to monocyte infiltration, must be carefully refined to ensure that these genes are physiologically and pathologically relevant, and importantly, implicated in the disease process. This is crucial for identifying viable new therapeutic targets. In this regard, scRNA‐Seq serves as a valuable complementary tool to help refine and validate the candidates by aligning them with the specific gene expression profiles of different cell populations involved in the disease. The integrated workflow (Figure 3D) is designed to enhance the specificity and relevance of potential therapeutic targets identified. Combining scRNA‐Seq with CRISPR‐MI could become a powerful tool to decode monocyte infiltration associated with abdominal aortic aneurysm. CRISPR‐MI is a highly flexible technique that can be applied to other monocyte‐related diseases, e.g., atherosclerosis, fatty liver disease, or cancers. In addition, CRISPR‐mediated transcriptional activation or inhibition screens can also be utilized in CRISPR‐MI. Here, we found 8 genes including *Trem2*, *Acp5*, *Tppp3*, *Ctsd*, *Tsc22d3*, *Tmem176b*, *Clec4d*, and *Klf9*, could be key genes regulating monocyte infiltraion. Although we mainly focus on *Trem2*, other genes also worth further investigation in future studies.

By combining scRNA‐Seq and CRISPR‐MI, we have identified *Trem2* as a regulator of monocyte infiltration and inflammation. The upregulation of *Trem2* expression has been consistently reported in multiple inflammatory diseases, including nonalcoholic steatohepatitis,^[^ ^85^ ^]^ atherosclerosis,^[^ ^86^ ^]^ and obesity.^[^ ^87^ ^]^ However, the biological function of *Trem2* in vascular diseases remains controversial. Global KO of *Trem2* reduced atherosclerosis in *ApoE* ^−/−^ mice;^[^ ^40^ ^]^ CX3CR1‐Cre^ER^ mediated myeloid selective *Trem2* KO reduced atherosclerosis in *Ldlr* ^−/−^ mice;^[^ ^41^ ^]^ *Trem2* global KO or *Trem2* KO bone marrow transplantation did not influence atherosclerotic plaque size but increased necrotic core of atherosclerotic lesion in *Ldlr* ^−/−^ mice;^[^ ^42^ ^]^ TREM2 agonist antibody (AL002a) increased atherosclerotic plaque size and stability.^[^ ^88^ ^]^ These conflicted results could be attributed to differences in KO strategies and disease models. TREM2 has been reported to be a receptor for ApoE,^[^ ^89^, ^90^ ^]^ so the results from *ApoE* ^−/−^ strain may differ from the *Ldlr* ^−/−^ strain. Irradiation before bone marrow transplantation cannot eliminate all macrophages in recipient mice,^[^ ^91^ ^]^ and CX3CR1‐Cre^ER^ cannot delete *Trem2* in CX3CR1^−^ macrophage. Soluble TREM2 and membrane‐bound TREM2 may also exert different biological functions,^[^ ^82^ ^]^ and agonist antibodies could only partially mimic TREM2 function in vivo. In our study, we used global *Trem2* KO mice to ensure *Trem2* was completely depleted. Because *Trem2* was exclusively expressed in myeloid cells in the aortic wall (Figure 3e), we consider it a good model to study the biological roles of *Trem2* in macrophage. It is noteworthy that it was reported *Trem2* KO might influence metabolism and plasma lipid profile.^[^ ^87^ ^]^ In our AAA model, *Trem2* KO or sTREM2 overexpression did not change lipid profile or body weight, excluding the potential confounding effects of *Trem2* KO on global metabolism. Recently, bone‐marrow transplantation also demonstrated that TREM2^+^ macrophages were key mediator of vascular damage and aneurysm development,^[^ ^92^ ^]^ in agreement with our findings.

Consistent with the CRISPR‐MI result, we demonstrated that *Trem2* was a negative regulator of adhesion molecules, chemotactic receptors, and cytokines in monocytes (Figure 5). In the in vivo study, we also confirmed that *Trem2* KO monocytes exhibited higher infiltration capacity (Figure 4). CRISPR‐MI was based on the physical presence of macrophages in the aortic wall, but not the biological function of the macrophage. Different macrophage clusters may play distinct roles in disease development, e.g., pro‐inflammation, pro‐resolution, or pro‐repair. As a result, the consequence of increased *Trem2* KO macrophage infiltration still requires biological confirmation.

Interestingly, although we found that *Trem2* KO promoted monocyte infiltration at the early time points (3 and 9 days) after AngII infusion, the number of macrophages in the aortic wall decreased in *Trem2* KO mice after 28‐day AngII infusion. We supposed that this was due to increased apoptosis of *Trem2* KO macrophage in the aortic wall. This phenomenon was also observed in the BMDM in vitro culture: *Trem2* KO BMDM began to undergo significant cell death after a 5‐day culture, corresponding to the monocyte‐macrophage transition stage. The relationship between macrophage apoptosis and AAA was not well‐studied yet. In atherosclerosis, it has been shown that in early atherosclerotic plaques, the selective removal of macrophages via macrophage‐specific death could have plaque‐stabilizing effects and reduced plaque inflammation.^[^ ^93^ ^]^ In the *Trem2* KO mice, we did not observe decreased monocytes in the circulation, so we supposed that *Trem2* might be disposable for monocyte differentiation and survival but critical for monocyte‐to‐macrophage maturation and mature macrophage survival. The mechanistic study revealed that *Trem2* KO abolishes the SYK activation, which is important for immune cell differentiation, proliferation, and survival.^[^ ^94^ ^]^ TREM2 binds to DAP12 and recruits SYK kinase to activate downstream molecules. TREM2/DAP12 crosstalk with M‐CSF/CSF1R pathway for macrophage differentiation and survival.^[^ ^94^ ^]^ In short, we found that TREM2 plays at least 2 distinct roles in monocyte/macrophage: one is to maintain monocyte quiescence in circulation, and the other one is to maintain macrophage survival in tissue.

In microglia, TREM2 inhibited inflammatory response and promoted phagocytosis. Anti‐TREM2^[^ ^95^, ^96^ ^]^ agonistic antibody or sTREM2 recombinant injection^[^ ^81^ ^]^ ameliorated the pathology in Alzheimer's disease, making them appealing new treatments. However, our study revealed that overexpression of sTREM2‐Fc chimeric protein could worsen AAA lesions in a mouse model. Additionally, other studies have shown that *Trem2* knockout decreases atherosclerotic lesions.^[^ ^40^, ^41^ ^]^ This raises concerns about using TREM2 as a therapeutic target in Alzheimer's disease, highlighting the need to avoid potential cardiovascular side effects of TREM2 agonist therapy. Conversely, TREM2 antagonists could potentially treat AAA, although neurological side effects must be considered.

## 4 Conclusion

In conclusion, our CRISPR‐MI identified that TREM2 was a negative regulator of monocyte infiltration. Loss of *Trem2* in monocytes increased the expression of adhesion molecules, chemotactic receptors, and cytokines via the ERK pathway. *Trem2* KO increased monocyte recruitment into the aorta in the AAA model. However, *Trem2* KO macrophage underwent prominent apoptosis in the aortic wall, and *Trem2* KO attenuated AAA phenotype after 28‐day AngII infusion, while sTREM2 overexpression aggravated AAA phenotype in the AngII infusion model. Thus, TREM2 is an important regulator of both monocyte extravasation and macrophage survival.

## 5 Experimental Section

### Mouse

*Trem2* knockout mice (C57BL/6J‐Trem2^em2Adiuj^/J) were purchased from The Jackson Laboratory (Strain #027 197). C57BL/6J‐Trem2^em2Adiuj^/J was a CRISPR/Cas9‐generated knockout mutant of the *Trem2* gene. The allele had an NHEJ‐generated 175 bp deletion that introduced a stop codon at amino acid 17.^[^ ^97^ ^]^ *Trem2* heterozygotes were bred with *Trem2* heterozygotes to generate *Trem2* WT and KO mice. Littermate controls were used in the study. The *Ldlr* knockout (Strain# 002 207), C57BL/6J (Strain# 000 664), Rosa26‐Cas9 (Strain# 028 555), and mTmG (Strain# 007 676) were purchased from The Jackson Laboratory. All animals were housed in a specific pathogen‐free animal facility on a 12 h light/12 h dark cycle. All animal work was performed according to protocol approved by the Institutional Animal Care and Use Committee at the University of Michigan (PRO00011743).

### Bone Marrow‐Derived Macrophage Culture and Adoptive Transfer

Bone marrow was flushed out from mouse femur and tibia as previously described.^[^ ^47^ ^]^ Bone marrow cells were cultured in a 15 cm petri dish (Nest Biotech) in IMDM (Gibco) supplemented with 10% FBS + 30 ng mL^−1^ M‐CSF (R&D system) for 5 days. BMDMs were washed with ice‐cold PBS and gently detached with Accutase (Innovative Cell Technologies, AT104). BMDMs were washed by PBS and resuspended in serum‐free RPMI1640 medium (Gibco). BMDMs were slowly injected into the tail vein of recipient mice.

### CRISPR‐MI

Lentivirus carrying sgRNA library was transduced into BMDMs from Rosa26‐Cas9 Tg mice 48 h after isolation with F108. The lentivirus dosage (30 MOI) was optimized to make sure that only 30–50% BMDMs were tdTomato^+^ to avoid two lentiviruses in the same cell. 22 h after infection, the BMDMs were collected and transferred into recipient mice. The recipient mice were injected with AAV‐PCSK9, fed with a Western diet (21 days), and infused with AngII (7 days) before BMDM transfer. Three days after the BMDM transfer, the aorta, heart, and liver were collected for analysis.

For CRISPR‐MI, the total BMDM number required was calculated as follows:

(1) $$\begin{array}{ccc} \mathrm{Total}\mathrm{cells}\mathrm{required} & = & \frac{\mathrm{sgRNA}\mathrm{number}\times \mathrm{coverage}}{\mathrm{Transfection}\mathrm{efficiency}}\\ & & =\frac{8\times 10^{4}\times 400}{0.4}=8\times 10^{7} \end{array}$$

For each mouse, this study could get ≈2 × 10^7^ BMDMs. As a result, to achieve 400 coverage of sgRNA, four mice were used as donors, and four mice were used as recipients in one replicate. The BMDMs were pooled before the transfer, and DNA from the same organs was pooled before PCR. 10% BMDMs were taken as background control before transfer. Two biological replicates were performed for the screen to check the repeatability of CRISPR‐MI.

DNAs were extracted with Blood & Cell Culture DNA Maxi Kit (Qiagen). sgRNAs were PCR amplified with sgRNA library NGS primers in Table S4 (Supporting Information). The PCR products were purified by gel extraction and sent to the University of Michigan Advanced Genomic Core for next‐generation sequencing. PCR products from BMDMs before transfer were used as background control. The alignment and quantification of sgRNAs after sequencing were conducted with the Rsubread R package (v2.9.5). The enrichment and statistical analysis were performed with MAGeCK tools (v0.5.9) with default parameters. Enriched genes were defined as that at least 2 out of 4 sgRNA were enriched (FDR < 0.05) in the aorta.

The selection of genes likely involved in macrophage infiltration was based on the following criteria: 1) CRISPR‐MI positive hits: genes with >= 2 gRNA enriched in aorta (FDR < 0.05), total 2516 genes. The data was analyzed with MAGeCK tool, and results were shown in Supplemental Table S1; 2) Genes differentially expressed in macrophage subpopulation: genes with absolute log2 fold change > 1 and adjusted p‐value <0.01 between different subpopulations, total 444 genes. The scRNA data was analyzed by Seurat R package with FindMarkers function with Wilcoxon Rank Sum test and default parameters; (3) Genes differentially expressed in macrophage under AAA versus Control condition: genes with absolute log2 fold change >1 and adjusted p‐value <0.01, total 74 genes. The data was analyzed by Seurat R package with FindMarkers function with Wilcoxon Rank Sum test and default parameters

### Mouse AAA Model

The angiotensin II (Ang II)‐induced mouse AAA model was generated in accordance with established protocols.^[^ ^98^ ^]^ Both male and female mice, aged 7–10 weeks as specified in the figure legends, were subjected to one of two treatments. They either received an intraperitoneal injection of 2×10^11^ genomic copies of adeno‐associated virus (AAV) encoding a gain‐of‐function mutation of mouse PCSK9 (AAV‐PCSK9.D377Y) from Penn Vector Core or an intravenous injection of an equal amount of either AAV‐Fc or AAV‐sTREM2‐Fc. The original plasmid for AAV‐sTREM2‐Fc was provided by Dr. Jiandie Lin from the University of Michigan, and the viruses were produced at the Vector Core facility at the University of Michigan. Post‐injection, the mice were transitioned to a Western diet containing 0.2% cholesterol by mass (Envigo, Cat#TD.88137). Following 2 weeks from the AAV administration, the mice were subcutaneously implanted with osmotic mini‐pumps (Alzet, Model #2004) to deliver Ang II (Bachem, Cat#4006 473) over 28 days at a rate of 1000–1500 ng kg^−1^ min^−1^, dosage specified in the figure legends. A control group was administered an equal volume of saline. The maximal external diameters of the suprarenal abdominal aortas were measured in situ, in a double‐blinded manner, using a digital caliper (Fisher Scientific, Cat# No. 14‐648‐17).

### Statistics

All measurements were taken from distinct samples. Statistics tests were performed in R (for RNA‐Seq and scRNA‐Seq) or GraphPad Prism version 10.1 (GraphPad Software, San Diego, CA). Unless indicated otherwise, values were presented as mean ± standard error of the mean (SEM). All data were tested for normality and equal variance. If the data passed those tests, the two‐tail Student *t*‐test was used to compare two groups. One‐way ANOVA or two‐way ANOVA followed by Holm‐Sidak post hoc analysis was used for comparisons among >2 groups. If the data did not pass those tests, Mann‐Whitney was used to compare the two groups. P‐value < 0.05 was considered statistically significant.

### Study Approval

Human sample collection was approved by the Ethics Committee of the Xiangya Hospital, Central South University (Approval Number: No.20220204348) and the Institutional Review Board (IRB number Hum00131275) from the Human Research Protection Program and Institutional Review Boards of the University of Michigan Medical School. All experimental procedures involving animals were conducted following the guidelines approved by the Institutional Animal Care & Use Committee (IACUC) at the University of Michigan.

## Conflict of Interest

The authors declare no conflict of interest.

## Author Contributions

H.L., C.X., Y.Z., J.S., C.Z., X.Z., G.Z., and Y.L. performed experiments and analyzed the results. H.L., C.X., Y.Z., J.Z., and E.C. wrote the manuscript. H.L., Y.D., Y.W., C.Z., Y.L., L.Z., Y.Y., B.L., S.D., L.Z., H.K., Z.W., W.J., H.L., J.L., Y.G., L.C., H.Z., J.W., J.L., L.Z., and J.Z. provided technical support. H.L., J.Z., and E.C. designed the research and discussed the results. The co‐first authors, H.L., C.X., and Y.Z. equally contributed to the data generation and analysis.

## Supporting information



Supporting Information

Supplemental Table 1

Supplemental Table 2

## Data Availability

The data that support the findings of this study are available in the supplementary material of this article.
