# Supplementary material for: CRISPR‐MI and scRNA‐Seq Reveal TREM2's Function in Monocyte Infiltration and Macrophage Apoptosis During Abdominal Aortic Aneurysm Development
Source: Adv Sci (Weinh). 2025 Oct 7;12(48):e12227. doi: 10.1002/advs.202412227 (PMC12752598; doi:10.1002/advs.202412227)
Supplement: Supplementary file 1 — Supporting Information [file ADVS-12-e12227-s002.docx]

Supporting Information

Title CRISPR-MI and scRNA-Seq reveal TREM2’s function in monocyte infiltration and macrophage apoptosis during abdominal aortic aneurysm development

*Haocheng Lu*, Chao Xue, Yang Zhao, Jinjian Sun, Changzhi Zhao, Xu Zhang, Guizhen Zhao, Yaozhong Liu, Hongyu Liu, Yongjie Deng, Ying Wang, Chi Zhang, Yingjie Liu, Linjun Zeng, Ying Yang, Bolun Li, Shusi Ding, Linkang Zhou, Henry Kuang, Zanxin Wang, Wenhao Ju, Haihuan Lin, Jie Lin, Yanhong Guo, Lin Chang, Hongmei Zhao, Jing Wang, Jiandie Lin, Lemin Zheng,, Y. Eugene Chen*, Jifeng Zhang**

Antibody and other reagents used in this study are listed in the Table S3 below.

**Table S3:** Antibody and other reagents used in this study

| **Regent or Resource** | **Vendor or Source** | **Identifier** |
| --- | --- | --- |
| **Antibody** | | |
| CD11b, PE | Tonbo Biosciences | 50-0112-U100 |
| CD45, EF450 | Invitrogen | 48-0451 |
| F4/80, APC | Tonbo Biosciences | 130‑102‑471 |
| CD16/CD32 (Fc blocker) | Invitrogen | 14-0161-85 |
| TREM2 (for BMDM) | Cell Signaling Technology | 76765 |
| β-Actin | Cell Signaling Technology | 3700 |
| pERK | Cell Signaling Technology | 4370 |
| ERK | Cell Signaling Technology | 5013 |
| Mac2 | Invitrogen | 14-5301-82 |
| PARP | Cell Signaling Technology | 9542 |
| Caspase3 | Cell Signaling Technology | 14220 |
| pSYK | Cell Signaling Technology | 2710 |
| SYK | Cell Signaling Technology | 97818 |
| TREM2 (for plasma Fc-Trem2 chimeric protein) | Invitrogen | MA5-28224 |
| CD3, PerCP-Cyanine5.5 | Invitrogen | 45-0031-82 |
| CD19, FITC | BD Pharmingen | 557398 |
| CD62L, PE | Biolegend | 104407 |
| TREM2 (for aorta IF) | Abclonal | A10482 |
| Cleaved Caspase 3 | Proteintech | 68773-1-Ig |
| CD206/MRC1 | Cell Signaling Technology | 91992S |
| **Reagent** | | |
| Synperonic F108 | Millipore Sigma | 07579-250G-F |
| Polybrene | Millipore Sigma | TR-1003-G |
| Retronectin | TaKaRa | T100A |
| PGE2 | Cayman Chemical | 14010 |
| Calcein AM | Cayman Chemical | 14948 |
| Recombinant Mouse M-CSF Protein | R&D systems | 416-ML-050 |
| GDC-0994 | Cayman Chemical | 21107 |
| LPS from E. coli O111:B4 | Sigma | L2630 |
| Lipofectamine RNAiMAX | ThermoFisher Scientific | 13778150 |
| jetPRIME | Polyplus | 101000046 |
| Endura Electrocompetent Competent Cells | Biosearch Technologies | 60242 |
| iQ SYBR Green Supermix | Bio-Rad | 1708880 |
| Angiotensin II | Bachem | 4006473 |
| Minipump | Alzet | 2004 |
| Ficoll-Paque media | Sigma | GE17-1440-03 |
| VivoTrack 680 NIR Fluorescent Imaging Agent | Fisher Scientific | 50-209-8510 |
| QuickExtrac DNA  Extraction Solution | Lucigen | QE09050 |
| **Cell** | | |
| Lenti-X 293T Cell Line | TaKaRa | 632180 |
| Raw264.7 | ATCC | TIB-71 |
| Mouse aortic endothelial cells (MAECs) | Cellbiologics | C57-6052 |
| **Mouse** | | |
| Rosa26-Cas9 | The Jackson Laboratory | 028555 |
| mTmG | The Jackson Laboratory | 007676 |
| Trem2 knockout | The Jackson Laboratory | 027197 |
| Ldlr knockout | The Jackson Laboratory | 002207 |
| C57BL/6J | The Jackson Laboratory | 000664 |
| **Plasmid** | | |
| psPAX2 | Addgene | 12260 |
| pMD2.G | Addgene | 12259 |
| LentiGuide Cherry | Addgene | 170510 |
| Mouse Cherry Brie Pooled Library | Addgene | Pooled Library #170511 |
| **Kit** | | |
| RNeasy Mini Kit | Qiagen | 74106 |
| SuperScript III kit | Thermo Fisher  Scientific | 18080051 |
| Q5 High-Fidelity PCR Kit | New England Biolabs | E0555L |
| Blood & Cell Culture DNA Maxi Kit | Qiagen | 13362 |
| Mouse Trem2 ELISA Kit | RayBiotech | ELM-TREM2-2 |
| Human TREM2 ELISA Kit | Abcam | ab224881 |
| FITC Annexin V Apoptosis Detection Kit I | BD Biosciences | 556547 |
| QIAGEN Plasmid Plus Maxi Kit | Qiagen | 12963 |
| Lenti-X™ qRT-PCR Titration Kit | TaKaRa | 631235 |

*Flow cytometry:* BMDMs were blocked with 1:100 Fc blocker (eBioscience, Cat#14–0161-85) in FACS buffer (0.5% BSA in PBS) for 5 min on ice and then incubated with fluorochrome-conjugated antibodies for additional 30 min. Cells were sent to the Flow Cytometry Core at the University of Michigan. Antibodies used for flow cytometry are listed in the antibody list. FlowJo software was used for data analysis.

*Cell culture:* Lenti-293T cells and Raw264.7 cells were cultured in DMEM (Gibco) with 10% FBS (Thermo Fisher Scientific).

*ScRNA-Seq and analysis:* Single-cell RNA sequencing data (scRNA-Seq) were kindly shared by the corresponding author of the published paper (Front Cardiovasc Med. 2021 Nov 24;8:753711)[1]. ScRNA-Seq data was analyzed in Seurat R package (v5.0.1). The marker genes for each macrophage sub population were defined as adjusted p-value < 0.01 and log2 fold change > 0.6. The enrichment analysis was conducted with GSEA 4.3.3.

*In vivo image:* Detached BMDMs were washed PBS 3 times and stained with VivoTrack 680 NIR Fluorescent Imaging Agent (0.05 mg/mL) for 15 min at room temperature, protected from light. Cells were washed with PBS containing 1% FBS to remove the labeling agent. Three days after the adoptive transfer, the aorta was imaged with LI-COR DLx Odyssey imaging system under a 700nm channel.

*sgRNA library:* The sgRNA library was purchased from Addgene (#170511). To amplify the library, the plasmids were transformed into Endura Electrocompetent Competent Cells (Biosearch Technologies)[2]. To achieve enough coverage, parallel transformations were performed, and the number of colonies reached 200-time coverage. The plasmids were extracted using Plasmid Plus Maxi Kit (Qiagen).

*Lentivirus package and infection:* To produce lentivirus, Lenti-X 293T cells were co-transfected with 8μg pMD2.G plasmid, 16μg psPAX2 plasmid and 24μg sgRNA plasmid per 15cm dish, with JetPRIME (PolyPlus) according to the manufacturer's instructions. Forty-eight hours and 96 hours after transfection, the medium was collected and filtered by 0.45μm low protein binding membrane (Millipore). The collected medium was purified by sucrose cushion ultracentrifuge with Sorvall WX ultracentrifuge and SureSpin 630 rotor at 160,000g and 4 ºC for 2h[3]. The lentivirus titration was determined with the Lenti-X qRT-PCR Titration Kit (TaKaRa). The pellets were resuspended in PBS and stored at -80 ºC. Forty-eight hours after bone marrow isolation, lentivirus was added into the BMDM medium with 10mg/mL F108. After 8 hours, BMDMs were changed to a fresh culture medium.

*CRISPR-Seq:* sgRNAs were cloned into the LentiGuide Cherry vector (Addgene) by T4 ligase (NEB). Lentivirus was packaged in Lenti-X 293T cells with helper plasmid co-transection. Lentivirus carrying sgRNA was transduced into BMDMs with F108. Seventy-two hours after infection, genomic DNA was extracted with QuickExtract DNA Extract Solution (Lucigen) and PCR amplified with Q5 High-Fidelity DNA Polymerase (NEB). PCR products were purified by gel extraction and sent to Massachusetts General Hospital CCIB DNA core for CRISPR amplicon sequencing (CRISPR-Seq) to determine genome editing efficiency. The sgRNA and PCR primers are listed in Table S4.

**Table S4:** sgRNA and primer sequence.

| **Gene** |  | **Sequence (5’-3’)** |
| --- | --- | --- |
| mCd300lf | gRNA | CCTGTGATCCAGAAGAGAAA |
|  | Forward primer | GTGACCCGGTGTGAGAAGAT |
|  | Reverse primer | ACCAGGGCTCTCCTGTCTTT |
| mLmnb1 | gRNA | GTCTTGACAAGTTCACATAA |
|  | Forward primer | CCCCAAGAGCATCCAATAAA |
|  | Reverse primer | GCCCCTTTCCAAACAGTACA |
| mMylip | gRNA | TTTCGGTGATGGCTCGGTAG |
|  | Forward primer | GCCCCTTTCCAAACAGTACA |
|  | Reverse primer | CAAGGCTAGGGGAAGTCACA |
| sgRNA library NGS | Forward | GAGGGCCTATTTCCCATGAT |
|  | Reverse | CGGTGCCACTTTTTCAAGTT |

*Digestion of aortic cells:* Mice were euthanized and perfused with ice-cold PBS containing 2% heparin through the left ventricle. Aortae were digested in a cocktail of 450 U/mL collagenase type I (Gibco, #17100–017), 125 U/mL collagenase type XI (Sigma-Aldrich, #C7657), 60 U/mL hyaluronidase (Sigma-Aldrich, #H3506), and DNase-I (Roche, 10104159001) at 37°C for 45 min. Suspensions were filtered by a 100 μm cell strainer to isolate single cells. Cells were further blocked in Fc blocker (eBioscience, Cat#14–0161-85) for 5 min on ice and then incubated with fluorochrome-conjugated antibodies for an additional 30 min. Cells were sent to the Flow Cytometry Core at the University of Michigan.

*Adhesion assay:* Mouse aortic endothelial cells (MAECs) (Cellbiologics, Cat#C57-6052) cultured in complete endothelial cell medium at (Cellbiologics, Cat#MM1168) confluence were treated with TNFα (10 ng/mL) for 12h, followed by washing with PBS and re-culturing in Opti-MEM medium (Gibco, 31985062) for an additional 6h. BMDMs from isolated Trem2 WT and KO mice were stained with 1uM Calcein A/M and then seeded onto the MAECs for 6h and visualized using a fluorescent microscope (Keyencen, BZ X800). The retention rate was quantified by normalizing the Calcium A/M positive cell numbers post-washing to the prewashing area in each well. The Fiji ImageJ analyzer was used for quantification.

*Migration assay:* Mouse aortic smooth muscle cells (MASMCs) were isolated from 8-week-old male wild-type C57BL/6 mice as previously described[4]. MASMCs were seeded in the lower chambers of a Corning Costar Transwell (6.5 mm diameter, 8.0 μm pore size, Thermo Fisher, Cat#07-200-174) plate. The cells were then incubated in fresh opti-MEM (Gibco, Thermo Fisher Scientific; 31985-070) with or without TNF-α (20 ng/mL) stimulation for 12 hours. The medium was subsequently changed to fresh opti-MEM. BMDMs were seeded in the upper chambers of the Transwells and co-cultured with MASMCs in the fresh opti-MEM. After 6 hours of co-culture, the BMDMs on the upper surface of the Transwell insert were removed by scraping with a cotton swab, and the membranes were fixed in methanol for 30 minutes before staining with 0.2% crystal violet (MilliporeSigma, C0775) for 30 minutes at room temperature. Images were captured by light microscopy, and the area of crystal violet-positive regions was calculated using ImageJ software.

*BMDM RNA-Seq and analysis:* RNA was collected from BMDMs of *Trem2* WT and *Trem2* KO mice and treated with RNase-free DNase I (QIAGEN, 79254) at room temperature for 10 min before sequencing. RNA was sent to the advanced genomic core at the University of Michigan for library preparation and sequencing. DESeq2 tool was used to perform differential expression analysis. In addition, KEGG enrichment analysis was performed using GSEA v4.3.2.

*BMDM treatment and ERK inhibitor:* Bone marrow cells were cultured in a differentiation medium for 5 days. Subsequently, these cells were treated for 6 hours with either 50 nM GDC-0994, an ERK inhibitor, or a control treatment. Following this, RNA was isolated to measure the expression of genes related to inflammation and adhesion using quantitative PCR (qPCR).

*History and IHC:* After euthanasia, the mice were perfused with ice-cold saline via the left ventricle, followed by fixation using 10% formalin. After imaging, the suprarenal portions of the aortas were carefully dissected and then embedded in paraffin for sectioning. Hematoxylin and eosin (H&E), elastin, and Mac2 immunohistochemistry (IHC) staining were performed by the In-Vivo Animal Core (IVAC) at the University of Michigan. Elastin degradation grade was assessed by quantification of elastin fiber fragmentation and loss in the aortic tissue sections[5]. Areas positive or negative for IHC 3,3'-Diaminobenzidine (DAB) staining were quantitatively analyzed using ImageJ software.

*Human plasma collection:* Human cohort 1: The human plasma used in this study was obtained from the Department of vascular surgery, Xiangya hospital of Central South University, Human Province, China. Human sample collection was approved by the Ethics Committee of the Xiangya Hospital, Central South University (Approval Number: No.20220204348). All participating patients and their families provided written informed consent. The AAA patients and healthy donors enrolled were matched for the ages of 47-78 years old. The exclusion criteria were a range of cardiovascular conditions, including various forms of aortic diseases except for AAA, heart failure (with preserved or reduced ejection fraction, right-sided, left-sided, congestive), arrhythmias (such as atrial fibrillation and atrial flutter), hypertensive heart disease. Blood samples were collected from these participants, and plasma sTREM assays were performed by researchers blinded to the patient groups.

Human cohort 2: The human plasma used in this study was obtained from the Cardiovascular Health Improvement Project (CHIP) core at the Frankel Cardiovascular Center (CVC) of the University of Michigan, under the approval of the Institutional Review Board (IRB number Hum00131275) from the Human Research Protection Program and Institutional Review Boards of the University of Michigan Medical School. All participating patients provided written informed consent. The inclusion criteria were patients aged 44-80 years, with and without abdominal aortic aneurysm (AAA). The exclusion criteria were a range of cardiovascular conditions, including various forms of cardiomyopathy (non-ischemic, dilated, unspecified), valve diseases (mitral, tricuspid, and aortic valve stenosis, regurgitation, or insufficiency), heart failure (with preserved or reduced ejection fraction, right-sided, left-sided, congestive), arrhythmias (such as atrial fibrillation and atrial flutter), hypertensive heart disease, heart valve disease, and history of myocardial infarction. Blood samples were collected from these participants, and plasma sTREM2 assays were performed by researchers blinded to the patient groups. To ensure comparability, demographic data, drug usage, and biochemical parameters were analyzed and compared across the different patient groups.

**Table S5.** Demographic data of human cohorts.

| Human Cohort 1 | | | |
| --- | --- | --- | --- |
| Parameter | Control | AAA | p-value |
| Gender (Male/Female) | 9/3 | 18/2 | 0.3377 |
| Age (year, mean±sd） | 63.58±8.084 | 68.6±6.939 | 0.0725 |
| Smoking (Y/N/Unknown) | 5/2/5 | 13/5/2 | 0.1655 |
| Hypertension (Y/N) | 5/7 | 13/7 | 0.1543 |
| Plasma total cholesterol (mmol/L, mean±sd） | 5.105±1.301 | 4.586±1.321 | 0.3107 |
| Plasma total triglycerides (mmol/L, mean±sd） | 1.794±1.338 | 1.973±1.579 | 0.7565 |
| Human Cohort 2 | | | |
| Parameter | Control | AAA | p-value |
| Gender (Male/Female) | 13/0 | 17/0 | 1 |
| Age (year, mean±sd） | 60.54±3.666 | 61.65±9.546 | 0.6953 |
| Smoking (Y/N) | 7/6 | 13/4 | 0.2553 |
| Hypertension (Y/N) | 6/7 | 4/13 | 0.2553 |
| Plasma total cholesterol (mg/dL, mean±sd） | 164.3±59.14 | 145.6±24.35 | 0.524 |
| Plasma total triglycerides (mg/dL mean±sd） | 130.3±94.73 | 124.8±33.82 | 0.9049 |

*sTREM2 measurement:* Plasma samples collected with EDTA2K were centrifuged to separate lipids and the white blood cell (WBC) clot. The clear phase was then diluted at a 1:10 ratio. Plasma sTREM2 levels were measured using the Mouse Trem2 ELISA Kit (RayBiotech, ELM-TREM2-2) or the Human TREM2 ELISA kit (Abcam, ab224881), following the manufacturer's instructions.

*Western blot:* Cells were lysed using freshly prepared RIPA buffer (lab prep) supplemented with protease (Roche, Cat# 11873580001) and phosphatase inhibitors (Roche, Cat# 4906845001). Protein extracts were then separated on SDS-PAGE gels and transferred to nitrocellulose membranes. These membranes were blocked with either 5% milk or BSA (for phosphorylated protein detection) for 1 hour at room temperature, followed by overnight incubation at 4°C with primary antibodies, and subsequent incubation with fluorescence-labeled secondary antibodies (LI-COR Biosciences) at room temperature for 1-2 hours, using dilutions ranging from 1:5,000 to 1:10,000. The secondary antibodies used included 680RD and 800CW Donkey anti-rabbit IRDye (Cat#926-68073 and Cat#926-32213), 680RD and 800CW Donkey anti-mouse IRDye (Cat#926-68072 and Cat#926-32212), and IRDye® 680RD and 800CW Streptavidin (Cat#926-68079 and Cat#926-32230). Membranes were scanned with the LI-COR DLx Odyssey imaging system, and quantification of protein bands was performed using LI-COR Empiria Studio software.

*RNA preparation and RT-qPCR analysis:* Total RNA was extracted from cells using RNeasy Mini Kit (QIAGEN), followed by reverse transcription with SuperScript III kit (Thermo Fisher Scientific) and random primers. mRNA was determined by a Real-Time PCR Detection System (Bio-Rad) using iQ SYBR Green Supermix (Bio-Rad). The mRNA level was normalized to the internal control, *Gapdh*, unless otherwise mentioned. The primers used are shown in Table S6.

**Table S6:** primer sequence for qPCR:

| **Gene** |  | **Primer Sequence (5’-3')** |
| --- | --- | --- |
| mSell | Forward | TACATTGCCCAAAAGCCCTTAT |
|  | Reverse | CATCGTTCCATTTCCCAGAGTC |
| mFut4 | Forward | ACGGATAAGGCGCTGGTACTA |
|  | Reverse | GGAAGCCATAGGGCACGAA |
| mPecam1 | Forward | GCCTCACCAAGAGAACGGAA |
|  | Reverse | ATTGGATGGCTTGGCCTGAA |
| mItgb2 | Forward | CAGGAATGCACCAAGTACAAAGT |
|  | Reverse | CCTGGTCCAGTGAAGTTCAGC |
| mItgal | Forward | CCAGACTTTTGCTACTGGGAC |
|  | Reverse | GCTTGTTCGGCAGTGATAGAG |
| mItgam | Forward | GAGGCCCCCAGGACTTTAAC |
|  | Reverse | CTTCTTGGTGAGCGGGTTCT |
| mItgb1 | Forward | ATGCCAAATCTTGCGGAGAAT |
|  | Reverse | TTTGCTGCGATTGGTGACATT |
| mItga4 | Forward | GATGCTGTTGTTGTACTTCGGG |
|  | Reverse | ACCACTGAGGCATTAGAGAGC |
| mCcl2 | Forward | GGTCTTCAGCACCTTTGAATG |
|  | Reverse | ATTAAGGCATCACAGTCCGAG |
| mCcl3 | Forward | TACAAGCAGCAGCGAGTACC |
|  | Reverse | CGTGGAATCTTCCGGCTGTA |
| mCcl4 | Forward | TCTGTGCTCCAGGGTTCTCA |
|  | Reverse | CTCACTGGGGTTAGCACAGA |
| mCcl5 | Forward | GCAAGTCTCCAATCTTGCA |
|  | Reverse | CTTCTCTGGGTTGGCACACA |
| mCcr2 | Forward | ATCCACGGCATACTATCAACATC |
|  | Reverse | CAAGGCTCACCATCATCGTAG |
| mCcr5 | Forward | TTTTCAAGGGTCAGTTCCGAC |
|  | Reverse | GGAAGACCATCATGTTACCCAC |
| mCx3cr1 | Forward | GAGTATGACGATTCTGCTGAGG |
|  | Reverse | CAGACCGAACGTGAAGACGAG |
| mIl1a | Forward | CGAAGACTACAGTTCTGCCATT |
|  | Reverse | GACGTTTCAGAGGTTCTCAGAG |
| mIl1b | Forward | GCAACTGTTCCTGAACTCAACT |
|  | Reverse | ATCTTTTGGGGTCCGTCAACT |
| mTnf | Forward | CATCTTCTCAAAATTCGAGTGACAA |
|  | Reverse | TGGGAGTAGACAAGGTACAACCC |
| mTrem2 | Forward | CAGCCCTGTCCCAAGCC |
|  | Reverse | CTTCTTCAGGAAGGCCAGCA |
| mIl6 | Forward | CTGCAAGAGACTTCCATCCAG |
|  | Reverse | AGTGGTATAGACAGGTCTGTTGG |
| mIl12b | Forward | GGAAGCACGGCAGCAGAATA |
|  | Reverse | AACTTGAGGGAGAAGTAGGAATGG |
| mIl10 | Forward | GCTCTTACTGACTGGCATGAG |
|  | Reverse | CGCAGCTCTAGGAGCATGTG |
| mCd86 | Forward | TGTTTCCGTGGAGACGCAAG |
|  | Reverse | TTGAGCCTTTGTAAATGGGCA |
| mCd80 | Forward | ACCCCCAACATAACTGAGTCT |
|  | Reverse | TTCCAACCAAGAGAAGCGAGG |
| mNos2 | Forward | GTTCTCAGCCCAACAATACAAGA |
|  | Reverse | GTGGACGGGTCGATGTCAC |
| mGapdh | Forward | AGGTCGGTGTGAACGGATTTG |
|  | Reverse | TGTAGACCATGTAGTTGAGGTCA |

*m indicates mouse

*Annexin V staining:* BMDMs isolated from Trem2 WT and Trem2 KO mice were stained with the FITC Annexin V Apoptosis Detection Kit I (BD Biosciences, Cat#556547). In general, the cells were washed with cold PBS, followed by suspension in a 1x binding buffer, and then labeled with FITC Annexin V and Propidium Iodide (PI) for 10 to 15 min at room temperature. FACS was performed at the University of Michigan core facility.

*siRNA transfection:* RAW 264.7 cells or BMDMs were transfected with 20nM Silencer Select Negative Control siRNA (siControl, Thermo Fisher Scientific, 4390843) or other siRNA. siRNA for mouse *Trem2* was purchased from Thermo Fisher Scientific (Assay id Thermo Fisher MSS294503), and the rest of the siRNAs were ordered from Dharmacon. siRNA target sequences are listed below. Lipofectamine RNAiMAX Reagent (Invitrogen, 13778150) was used for transfection according to the manufacturer's instructions.

**Table S7**: siRNA sequence:

| **Gene** | **Target Sequence (5’-3')** |
| --- | --- |
| mAbhd6 | GCACUGAUAAGAAUCUAUU |
| mAscl4 | CUUCAUUGGUUCCGAGUUU |
| mChrna9 | GGAAAAUACUACAUAGCUA |
| mDusp28 | GAACUACGCGUACCCGUGU |
| mMicu2 | GAAAGAGCCUGGCGUUAAC |
| mGpr137b | GAUUAUGACUGGUACAAUG |
| mGpr37l1 | UAAACUAGCUGUCAUCUGG |
| mSp6 | GGACAUGUCACACCACUAU |
| mMarveld1 | GUAGAAGGCAAACGGGUCU |
| mTmem178 | GCACAGCAAUCAAGUACCA |
| mZnrf1 | CUGCAUAGACUCAUGGUUU |

*m indicates mouse

*ELISA of conditioned medium:* BMDMs isolated from Trem2 WT and Trem2 KO mice were treated with 1µg/mL LPS for 24 hours. Conditioned media from both treatment and non-treatment groups were collected. ELISA for IL6, IL-10, IL12p40, TNFα, CCL2, CCL3, CCL4, CCL5, VEGF, and PDGF-BB were performed by the Immunology Core at the University of Michigan.

*Plasma biochemistry:* Plasma total cholesterol (TC) and triglyceride (TG) levels were measured using enzymatic-based assay kits from Fujifilm Wako Diagnostics, specifically the Colorimetric-based Cholesterol E assay (Cat#999-02601) and the Triglyceride assay (Cat#290-63701).

*Blood pressure:* Blood pressure was non-invasively monitored using a tail-cuff method with the Visitech BP-2000 system.

*PBMC isolation:* Fresh blood was collected into BD Microtainer Tubes with K2EDTA, mixed immediately to prevent aggregation, and further supplemented with 20 μL of 0.5M EDTA. The blood was then diluted with an equal volume of DPBS. This diluted sample was carefully layered over 3 mL of Ficoll-Paque media (Sigma, Cat#GE17-1440-03) in a 15 mL centrifuge tube and centrifuged at 400 g for 40 min at 18°C–20°C without braking. The plasma-containing upper layer was removed, leaving the mononuclear cell layer undisturbed. The mononuclear cells were then transferred to a sterile centrifuge tube, diluted with three volumes of DPBS, and resuspended by gentle pipetting. After centrifugation at 400-500 x g for 15 min at 18°C–20°C, the supernatant was discarded, and cells were resuspended in 3 mL of balanced salt solution. For further RBC removal, 1 mL RBC lysis buffer was added, incubated for 10 min at room temperature, and halted with 10 mL balanced salt solution.

*Complete blood count and differentiation:* Fresh blood was collected into BD Microtainer Tubes with K2EDTA, mixed immediately to prevent aggregation, and further supplemented with 20 μL of 0.5M EDTA. Blood was kept at 4°C without direct exposure to ice to avoid hemolysis. A complete blood count with differentials was performed by the IVAC at the University of Michigan.

*Peritoneal macrophage isolation:* Primary peritoneal macrophages were isolated as previously published [6]. Thioglycollate (3%, w/v) in 2 ml sterile saline was administered intraperitoneally. After 96 h, mice were euthanized by CO2 overdose, and peritoneal cells were collected by washing the peritoneal cavity with 5 mL PBS twice.

*Human AAA tissue RNA-Seq and weighted gene co-expression network analysis (WGCNA):* The study was approved by the Institutional Review Board (IRB number Hum00131275) from the Human Research Protection Program and Institutional Review Boards of the University of Michigan Medical School. All participating patients provided written informed consent. Abdominal aortic tissue was surgically removed during open repair for abdominal aortic aneurysms. After excision, samples were maintained in a sterile environment, sectioned into 0.5 cm x 0.5 cm pieces, and submerged in RNAlater (Invitrogen, AM7020), where they remained at room temperature for 1-3 days. Subsequently, the RNAlater was removed, and the samples were preserved at -80°C. Tissues were then cryopulverized using a Covaris CP02 instrument (500001), and a portion of the pulverized tissue was utilized for total RNA isolation using Trizol (Invitrogen, ILT15596018) and RNeasy Mini Kit (Qiagen, 74106). Briefly, the RNA in the aqueous phase from the Trizol extraction was transferred to a new tube and mixed with an equal volume of 70% ethanol before processing on an RNeasy Mini kit column according to the manufacturer’s instructions. RNA was eluted in 50µl of RNase-free water. Concentrations were measured using the Qubit RNA Broad Range kit (Thermo Scientific, Q1021), followed by RNA integrity (RIN) evaluation by RNA TapeScreen on the Agilent 2200 TapeStation. RNA samples with a RIN > 7 were used for library preparation. The library was prepared using the SMART-Seq v4 Ultra Low Input RNA Kit (TaKaRa, 63489) and Nextera XT (Illumina, FC-131), followed by PE100 sequencing on the NovaSeq platform at MedGenome. Quality control was performed using FastQC, and alignment to the human genome hg38 was carried out using STAR. RNA quantification was performed using RNASeQC 2.4.2, and count normalization was conducted using DESeq2. The count matrices, filtered by an average abundance greater than 10, were analyzed using the R package WGCNA for co-variation analysis, clusterProfiler for intra-module enrichment analysis, and ggplot2 for plotting.

**Reference**

[1] B. Li, X. Song, W. Guo, et al., *Frontiers in cardiovascular medicine* **2021**, *8*, 753711, <https://doi.org/10.3389/fcvm.2021.753711>.

[2] G. Toda, T. Yamauchi, T. Kadowaki, K. Ueki, *STAR protocols* **2021**, *2* (1), 100246, <https://doi.org/10.1016/j.xpro.2020.100246>.

[3] G. Tiscornia, O. Singer, I. M. Verma, *Nat Protoc* **2006**, *1* (1), 241, <https://doi.org/10.1038/nprot.2006.37>.

[4] M. Hamblin, L. Chang, Y. E. Chen, in **2014**.

[5] H. Lu, J. Sun, W. Liang, et al., *Circulation* **2020**, *142* (5), 483, <https://doi.org/10.1161/circulationaha.119.044803>.

[6] H. Wang, Y. Guo, H. Lu, et al., *Cardiovascular research* **2022**, *118* (2), 475, <https://doi.org/10.1093/cvr/cvab027>.

**Supplemental figures and figure legends**


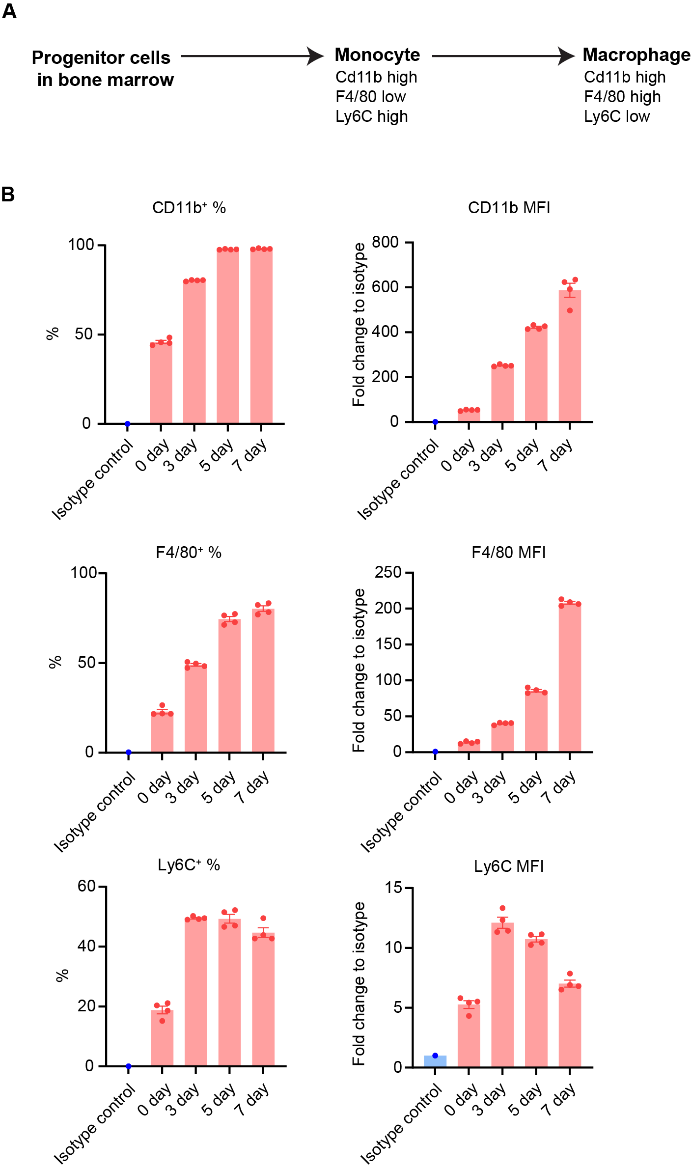
Figure S1

**Figure S1. Differentiation of BMDM *in vitro*.**

**A**, Schematics of marker gene expression at different stages of BMDM differentiation. **B**, BMDMs at different times after isolation were collected for staining and flow cytometry analysis (n=4). Data are presented as mean±SEM. MFI, mean fluorescence intensity.


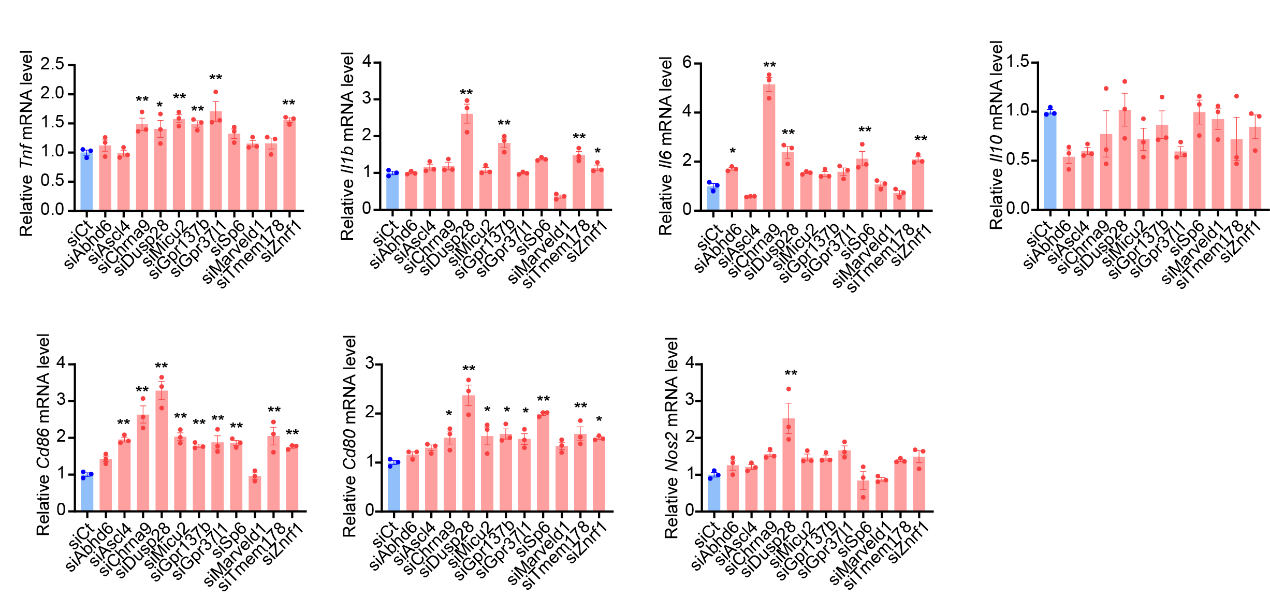
 Figure S2

**Figure S2. Validation of top hits in CRISPR-MI.**

Raw264.7 cells were transfected with siRNA negative control (siCt) or siRNA targeting genes with Lipofectamine RNAiMAX. 72h after transfection, cells were harvested for qPCR analysis (n=3). Data are presented as mean±SEM. One-way ANOVA was used. *, p <0.05; **, p<0.01.


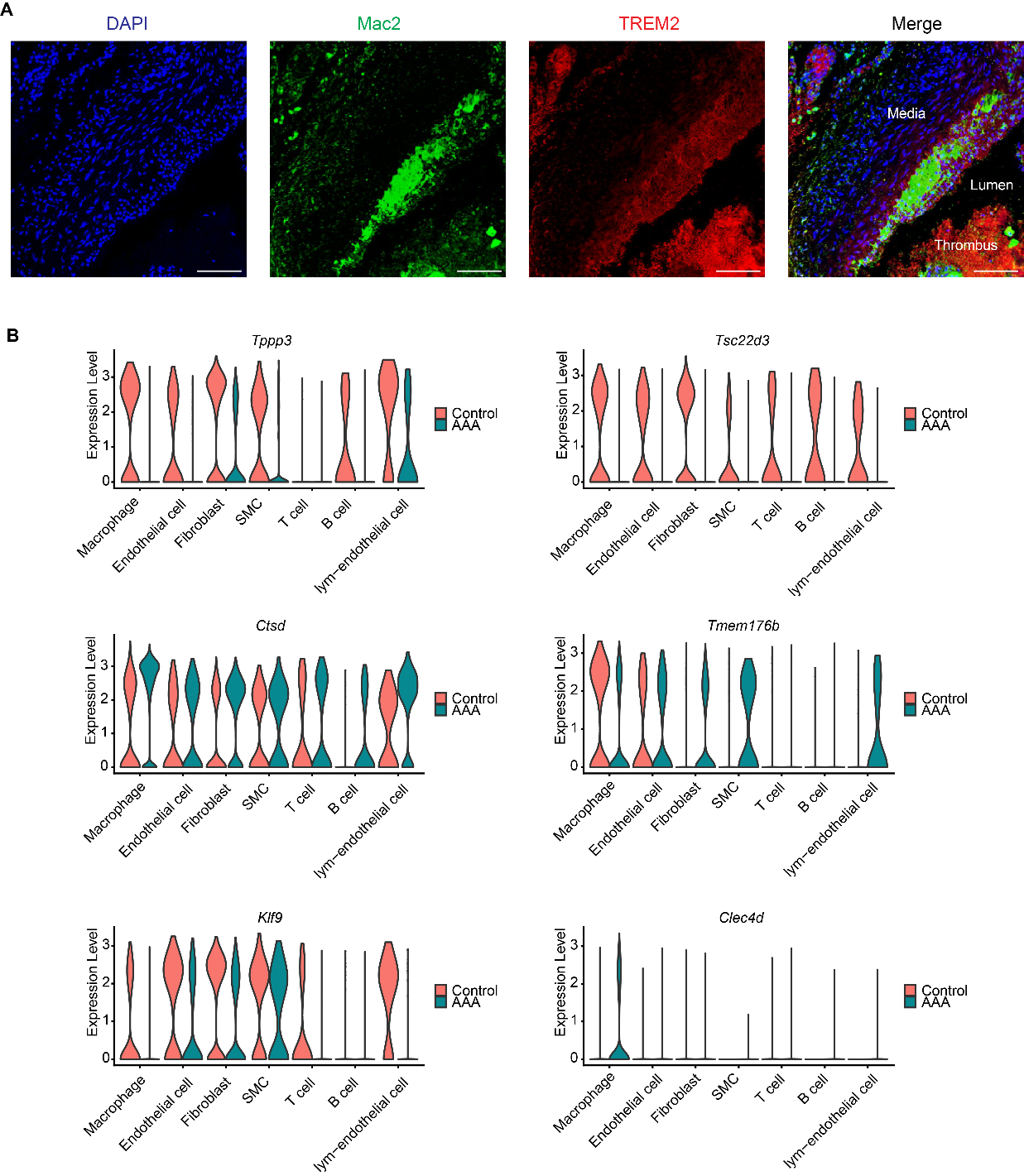
Figure S3

**Figure S3. Expression profiles of genes identified by CRIPSR-MI and scRNA-Seq. A,** TREM2, Mac2 (macrophage marker) expression in mouse aorta (after 28-day AngII infusion) were determined by immunofluorescence. Scale bar = 100μm **B,** Violin plot showing *Tppp3*, *Tsc22d3*, *Ctsd*, *Tmem176b*, *Klf9*, and *Clec4d* expression in each cluster in scRNA-Seq of mouse aorta.


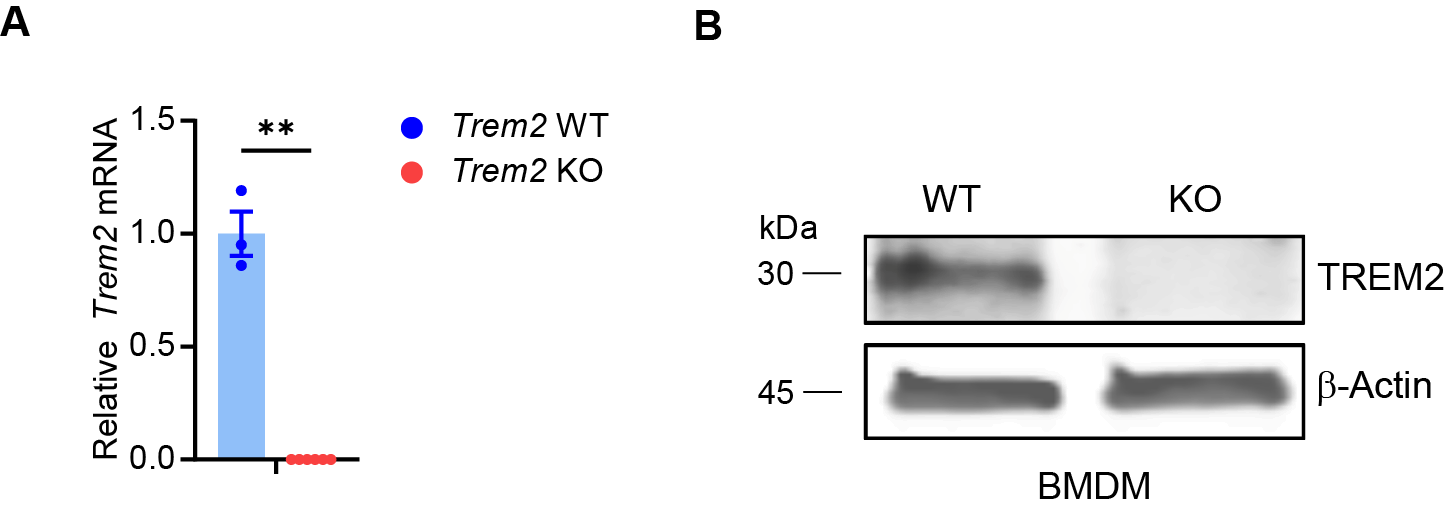
 Figure S4

**Figure S4. Validation of *Trem2* KO mice.**

BMDMs from *Trem2* WT and KO mice were harvested for qPCR (n=3, 5) and Western blot. Data are presented as mean±SEM. Unpaired t-test was used. **, p<0.01.

Figure S5

**
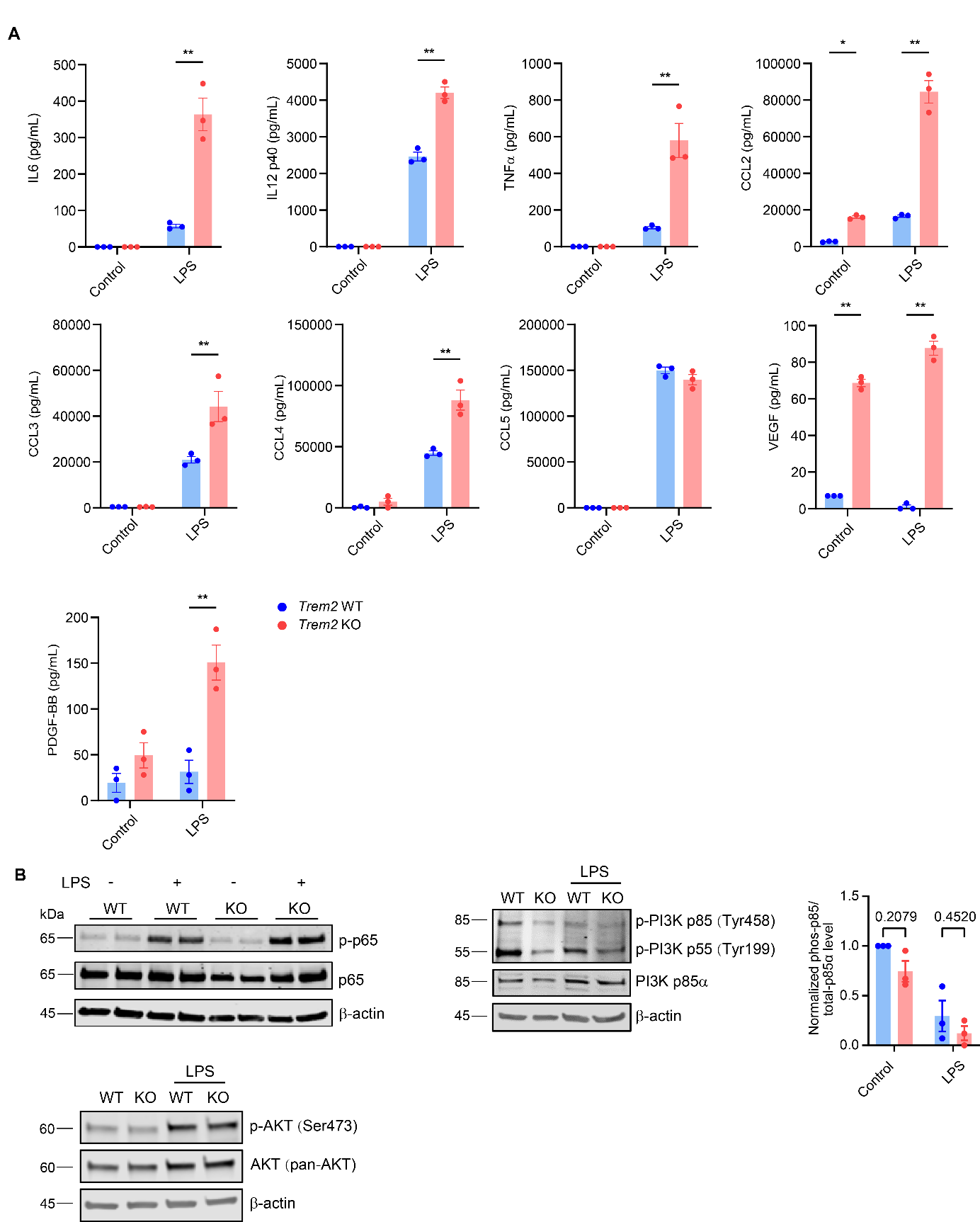
Figure S5. *Trem2* KO increases the secretion of cytokine and chemotactic molecules.**

**A**, BMDMs from *Trem2* WT and KO mice were treated with/without LPS (1μg/mL) for 24 hours. The conditioned medium was collected for ELISA (n=3). **B**, BMDMs from *Trem2* WT and KO mice were treated with/without LPS (1μg/mL) for 30 min and harvested for Western blot. Data are presented as mean±SEM. Two-way ANOVA was used. *, p <0.05; **, p<0.01.

Figure S6


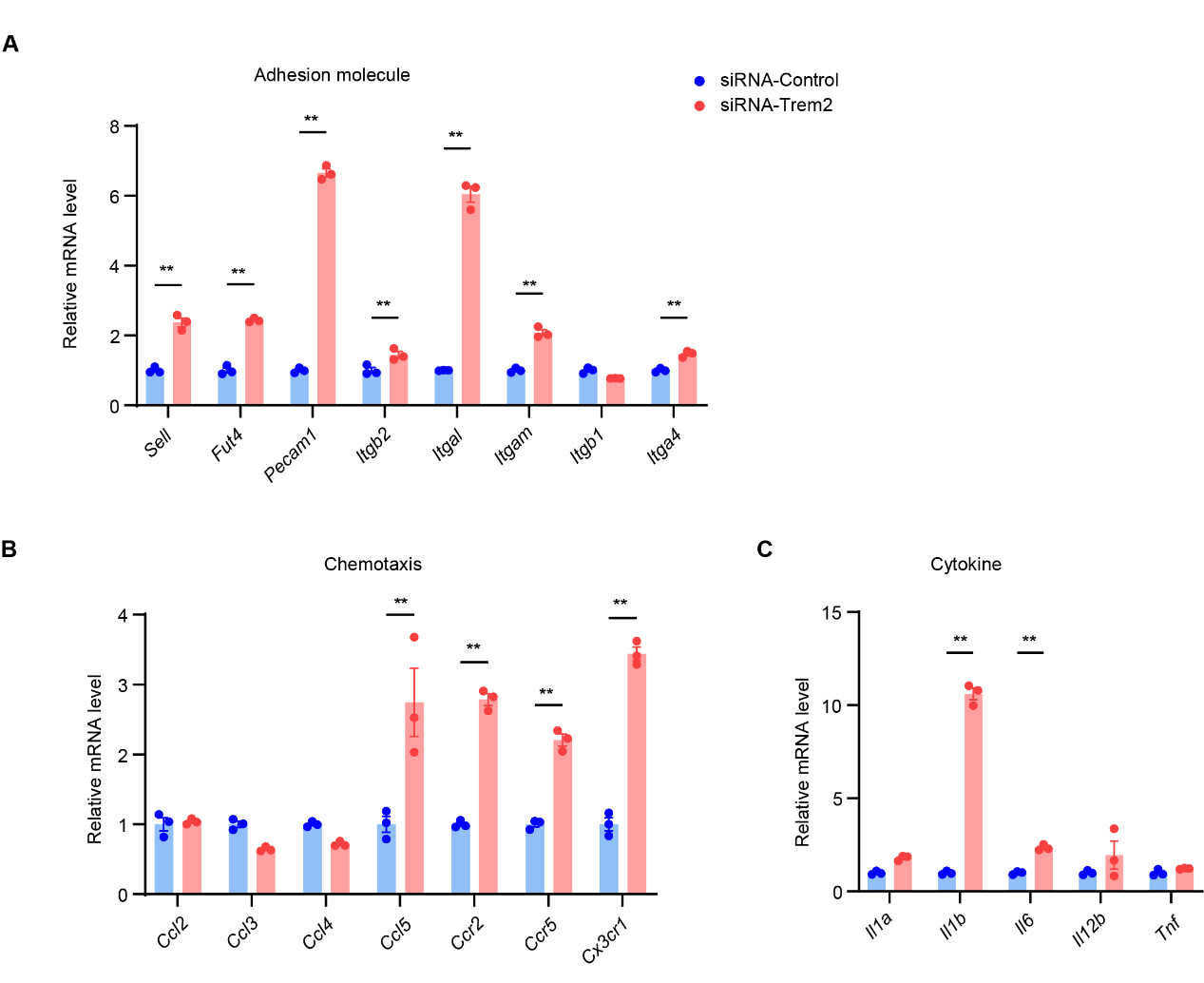


**Figure S6. *Trem2* transient knockdown increased the expression of genes involved in monocyte adhesion, chemotaxis, and inflammation.**

**A-C**, BMDMs were transfected with siRNA negative control (siCt) or siRNA targeting *Trem2* with Lipofectamine RNAiMAX. 72h after transfection, cells were harvested for qPCR analysis (n=3). Data are presented as mean±SEM. Two-way ANOVA was used. *, p <0.05; **, p<0.01.

Figure S7


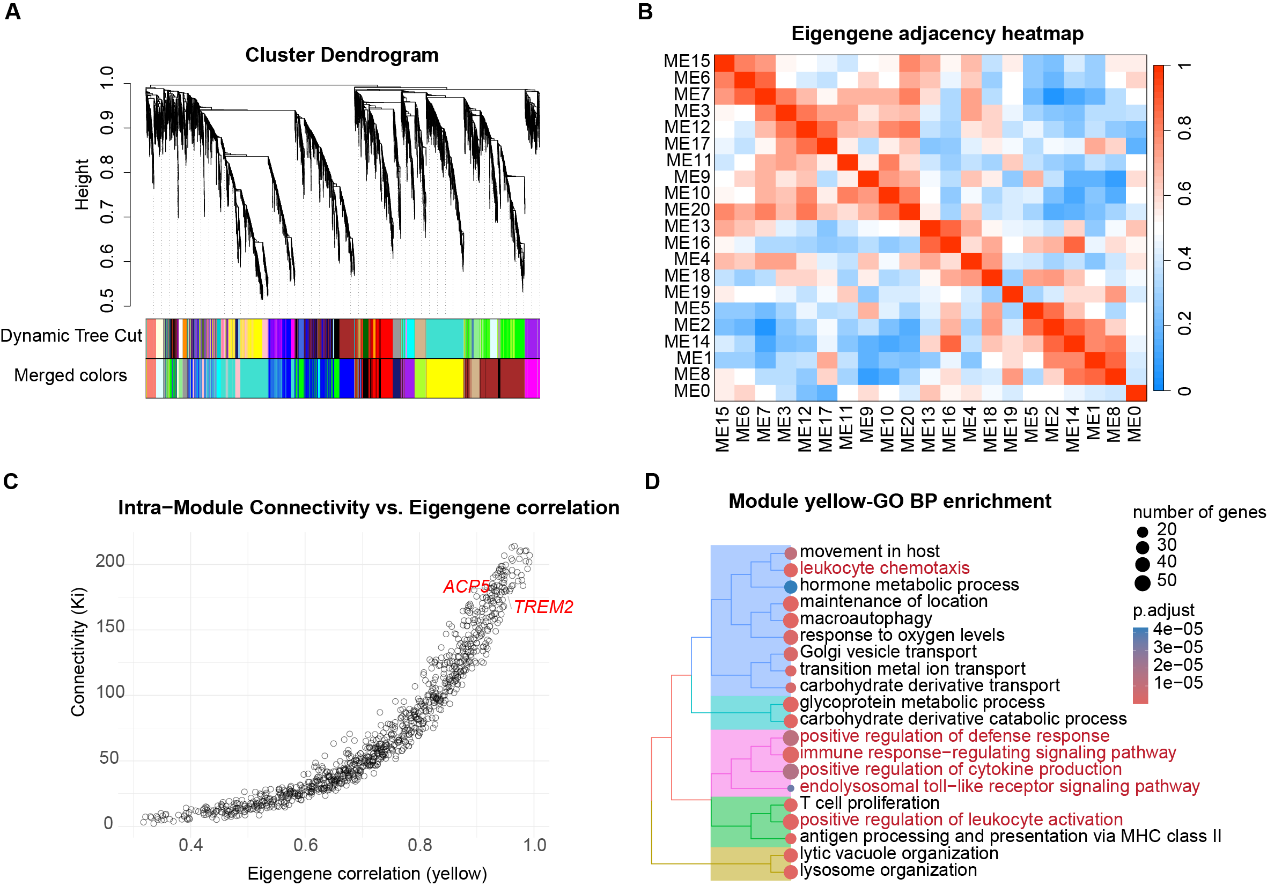


**Figure S7. *TREM2* is highly correlated with genes involved in leukocyte extravasation and inflammation in human AAA RNA-Seq data.**

We performed WGCNA for the top 10,000 most variant genes (Dseq2 normalized count > 10) extracted from 17 human patient RNA-Seq data, resulting in a total of 21 co-expression modules (power>0.9). **A,** Cluster dendrogram: Each color represents one specific co-expression module. In the colored rows below the dendrogram, the two rows represent the original modules and merged modules. **B**, Eigengene adjacency heatmap of different modules. **C**, *TREM2* was a hub gene in Module 4/Yellow, ranked 31/957 by intra-module connectivity. **D**, The Gene Ontology Biological Pathway enrichment of gene highly correlated in Module 4/Yellow module. The red text indicated pathways related to leukocyte extravasation and inflammation.

Figure S8


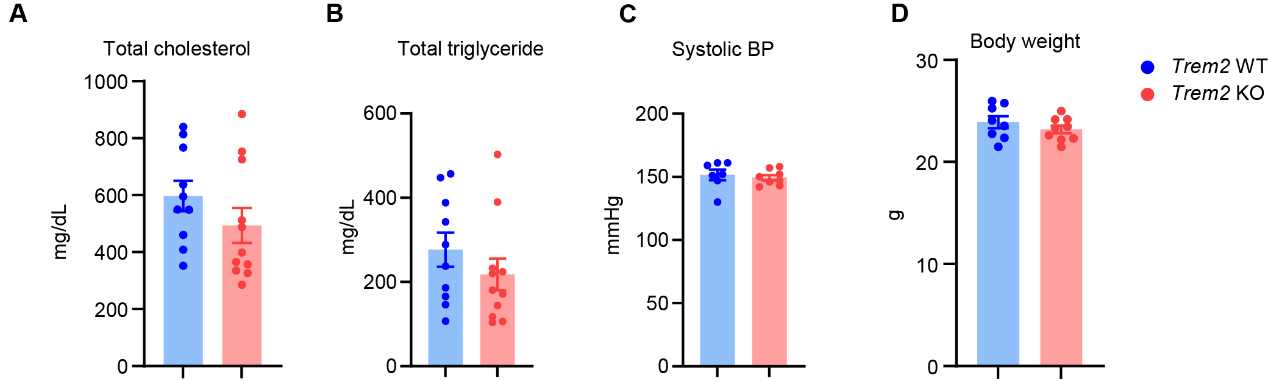


**Figure S8. *Trem2* KO did not influence plasma lipid, systolic blood pressure, and body weight in the AAA mouse model.**

**A-C,** Male *Trem2 WT and KO* mice were injected with AAV-PCSK9 and fed with a Western diet for 2 weeks, followed by saline or AngII infusion for 4 weeks. Plasma total cholesterol (A), total triglycerides (B), systolic BP (C), and body weight (D) were determined (n=10, 11). Data are presented as mean±SEM. Unpaired t-test was used.

Figure S9


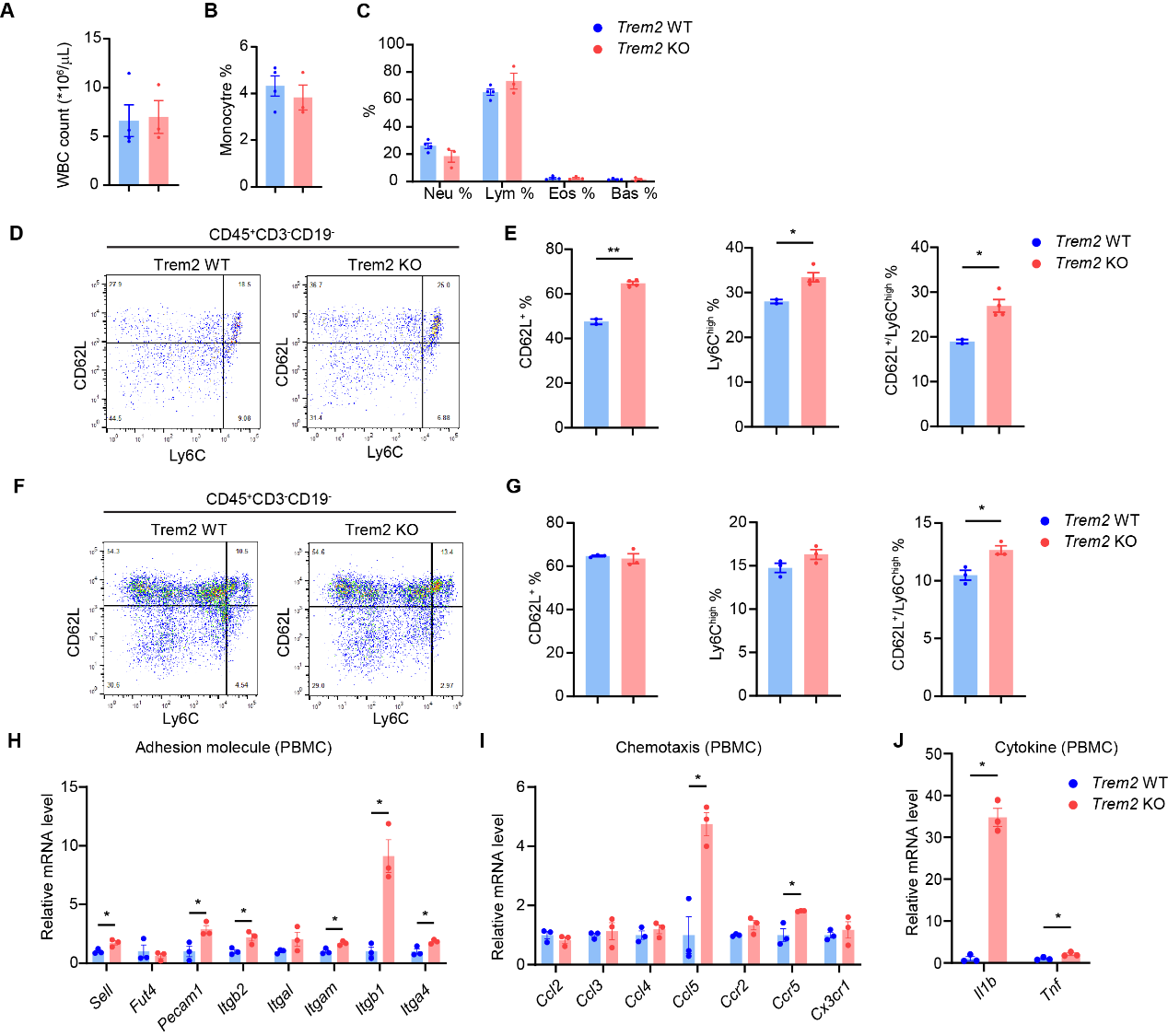


**Figure S9. *Trem2* KO increased pro-inflammatory monocytes in circulation.**

**A-C**, Blood was collected from *Trem2* WT and KO mice and underwent WBC count (A) and differential analysis (B-C) (n=4, 3). **D-G**, Peripheral blood mononuclear cells (PBMCs) were isolated from Trem2 WT and KO mice (D-E, n=2, 4) or *Trem2* WT and KO after AAA induction (F-G, n=3). PBMCs were stained and analyzed by flow cytometry. **H-J**, PBMCs from *Trem2 WT and KO* were collected for qPCR analysis (n=3). Data are presented as mean±SEM. Two-way ANOVA was used for C, H, I, and J. Unpaired t-test was used for A, B, E, G. *, p <0.05; **, p<0.01.

Figure S10


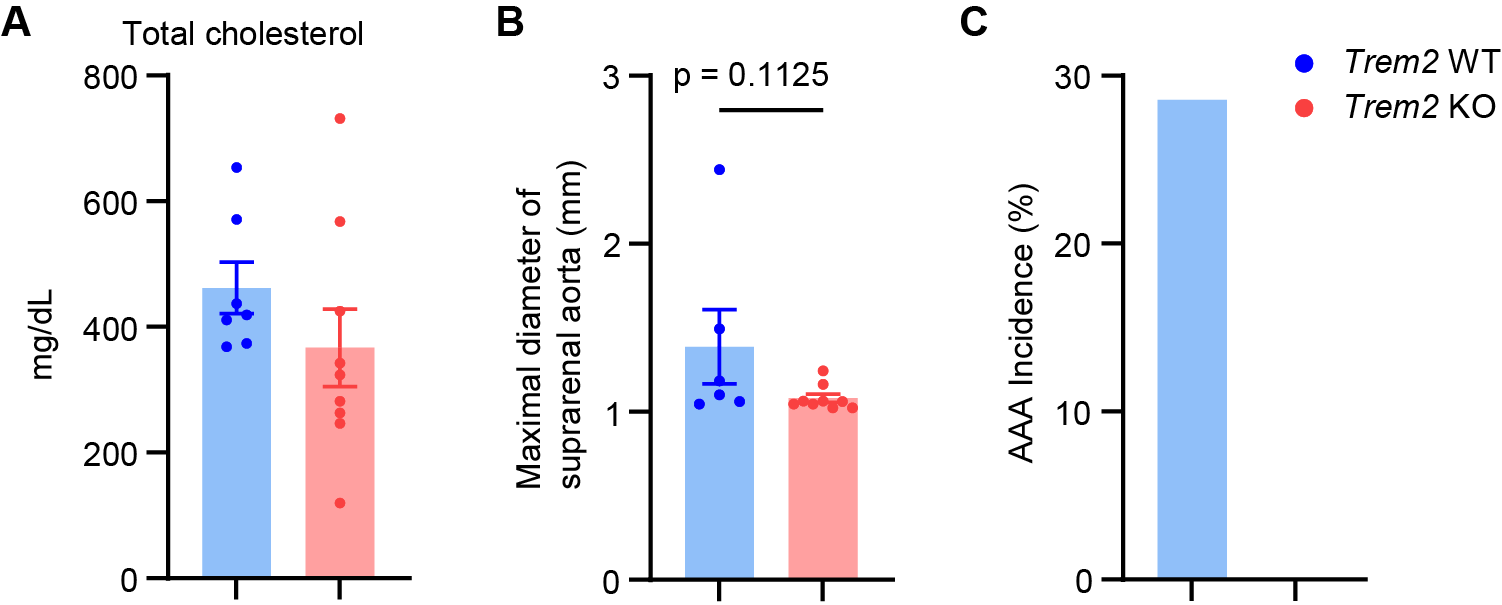


**Figure S10. Effect of *Trem2* KO on AAA development in female mice.**

**A-C,** Female *Trem2 WT and KO* mice were injected with AAV-PCSK9 and fed with a Western diet for 2 weeks, followed by saline or AngII infusion for 4 weeks. Plasma total cholesterol (A), maximal diameter of the suprarenal aorta (B), and AAA incidence (C) were determined (n=7, 9). Unpaired t-test was used for A-B.

**
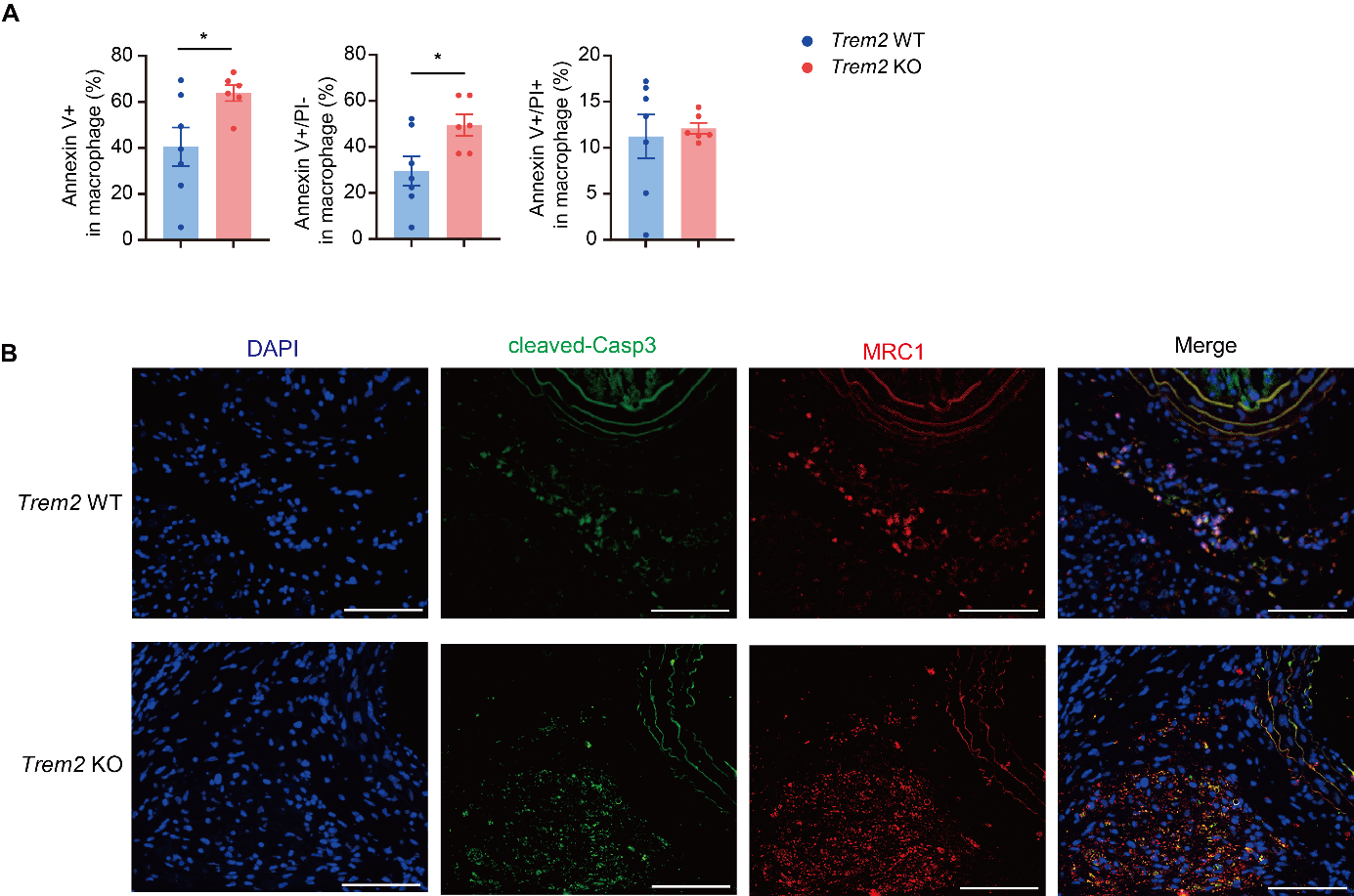
**Figure S11

**Figure S11. *Trem2* KO promotes macrophage apoptosis.**

**A**, *Trem2* WT and KO mice were injected with AAV-PCSK9 and fed with a Western diet for 2 weeks, followed by saline or AngII infusion (1500ng/kg/min) for 9 days. **A,** The aorta was digested and stained with CD45, CD11b, and Annexin V-FITC, followed by cyto flowmetry to determine apoptotic cells. **B,** The aorta was stained for cleaved-caspase3 and MRC1. Scale bar = 100μm. Unpaired t-test was used for A. **, p<0.01.


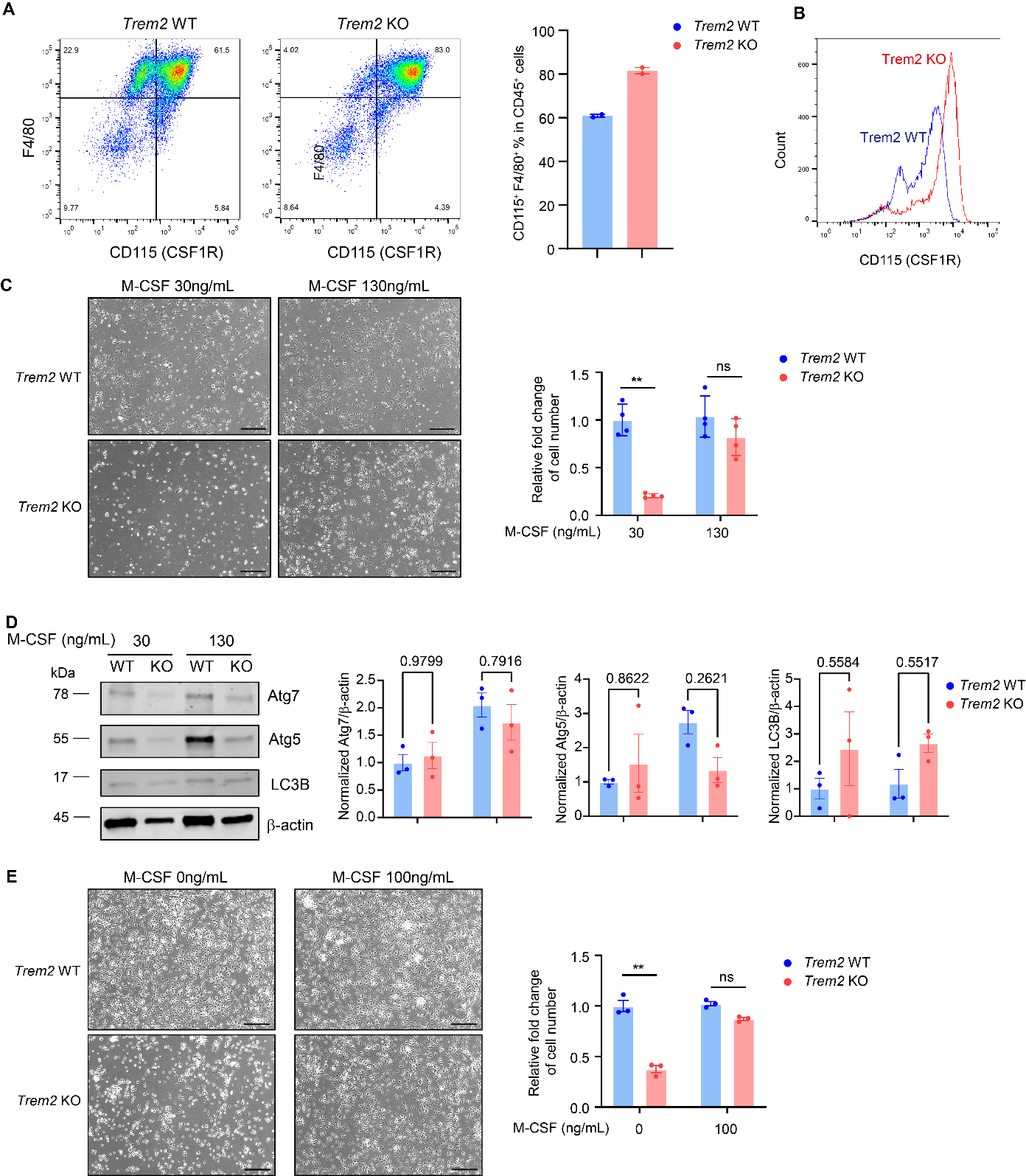
Figure S12

**Figure S12. *Trem2* KO promotes macrophage apoptosis.**

**A-B,** BMDMs from *Trem2* WT and KO mice were cultured for 5 days, followed by staining and flow cytometry analysis (n=2).**C-D**, BMDMs from *Trem2* WT and KO mice were cultured in BMDM medium (containing M-CSF 30ng/mL) for 3 days. On day 4, BMDMs were kept in BMDM medium (30ng/mL group) or treated with an additional 100ng/mL M-CSF (130ng/mL group). Bright field images were taken (**C**) and protein was harvest for Western blot on day 7 (**D). E**, Peritoneal macrophage was isolated from *Trem2* WT and KO mice. After harvesting, cells were treated with 0ng/mL or 100ng/mL M-CSF. Bright field images were taken after 24 hours. Two-way ANOVA for C, D, and E. **, p<0.01.


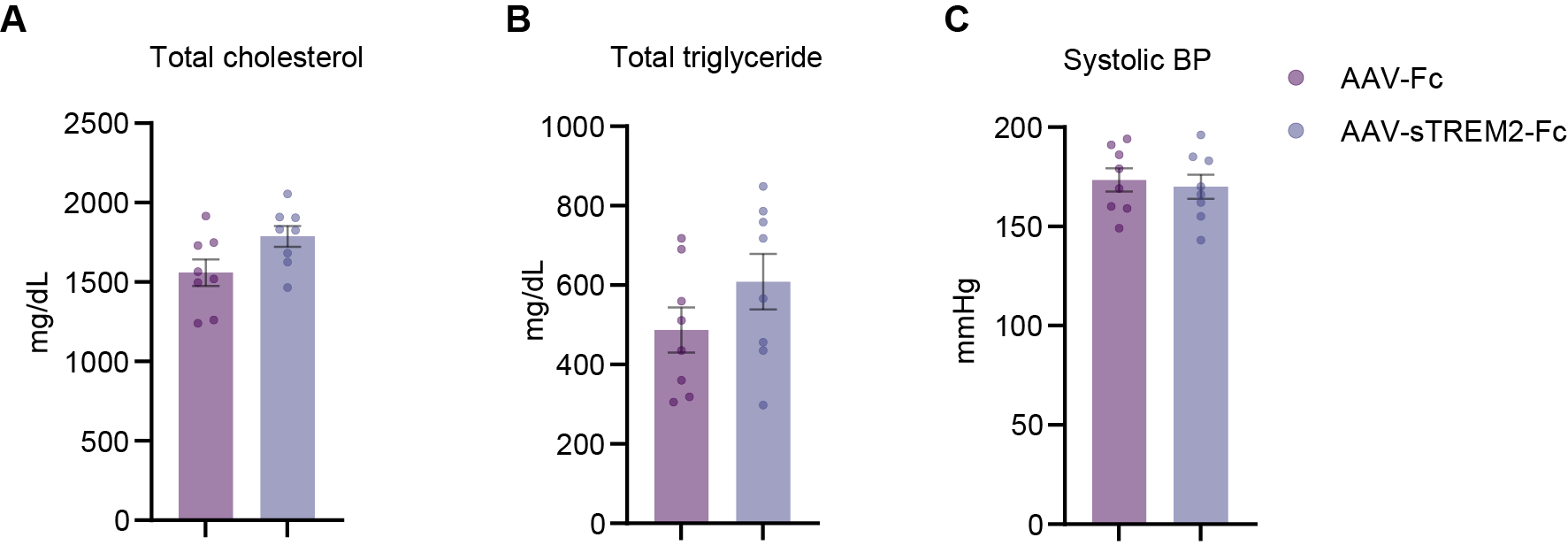
 Figure S13

**Figure S13. sTREM2 overexpression did not influence plasma lipid and systolic blood pressure in the AAA mouse model.**

**A-C,** *Ldlr* KO mice were injected with AAV-Fc or AAV-sTREM2-Fc and infused with AngII for 4 weeks (as in Figure 8B). Plasma total cholesterol (A), total triglycerides (B), and systolic BP (C) were determined (n=8). Unpaired t-test was used.

**Supplemental Table legends**

**Table S1. CRISPR-MI results.**

The CRISPR-MI results of genes enriched in the aorta, analyzed by MAGeCK. Positive RRA score, robust ranking aggregation (RRA) score of positive selection.

**Table S2. RNA-Seq results of *Trem2* KO vs *Trem2* WT BMDM.**

Comparison of mRNA expression between BMDM from Trem2 KO vs Trem2 WT mouse. baseMean: mean of normalized counts for all samples; lfcSE: standard error; stat: Wald statistic; padj: BH adjusted p-values.
